# Surface radio-mineralisation mediates chelate-free radiolabelling of iron oxide nanoparticles†
†Electronic supplementary information (ESI) available. See DOI: 10.1039/c8sc04895a


**DOI:** 10.1039/c8sc04895a

**Published:** 2019-01-09

**Authors:** P. Stephen Patrick, Lara K. Bogart, Thomas J. Macdonald, Paul Southern, Michael J. Powell, May Zaw-Thin, Nicolas H. Voelcker, Ivan P. Parkin, Quentin A. Pankhurst, Mark F. Lythgoe, Tammy L. Kalber, Joseph C. Bear

**Affiliations:** a Centre for Advanced Biomedical Imaging (CABI) , Department of Medicine , University College London , London WC1E 6DD , UK . Email: peter.patrick@ucl.ac.uk; b School of Life Science, Pharmacy & Chemistry , Kingston University , Penrhyn Road , Kingston upon Thames , KT1 2EE , UK . Email: J.Bear@kingston.ac.uk; c Materials Chemistry Centre , Department of Chemistry , University College London , 20 Gordon Street , London , WC1H 0AJ , UK; d UCL Healthcare Biomagnetics Laboratory , 21 Albemarle Street , London , W1S 4BS , UK; e Monash Institute of Pharmaceutical Sciences , Monash University , Parkville , Australia; f Commonwealth Scientific and Industrial Research Organisation (CSIRO) , Clayton , Australia

## Abstract

We introduce the concept of surface radio-mineralisation (SRM) to describe the chelate-free radiolabelling of iron-oxide and ferrite nanoparticles. We demonstrate the effectiveness of SRM with both ^111^In and ^89^Zr for bare, polymer-matrix multicore, and surface-functionalised magnetite/maghemite nanoparticles; and for bare Y_3_Fe_5_O_12_ nanoparticles. By analogy with geological mineralisation (the hydrothermal deposition of metals as minerals in ore bodies or lodes) we demonstrate that the heat-induced and aqueous SRM process deposits radiometal-oxides onto the nanoparticle or core surfaces, passing through the matrix or coating if present, without changing the size, structure, or magnetic properties of the nanoparticle or core. We show in a mouse model followed over 7 days that the SRM is sufficient to allow quantitative, non-invasive, prolonged, whole-body localisation of injected nanoparticles with nuclear imaging.

## Abstract 2

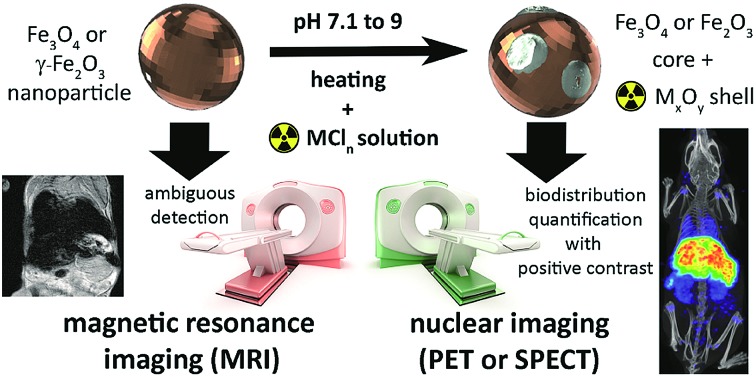
Mineralisation of radio-metals onto the surface of iron oxide nanoparticles simplifies radiolabelling, enabling quantification of their bio-distribution with nuclear imaging.

## Introduction

**Fig. 1:**
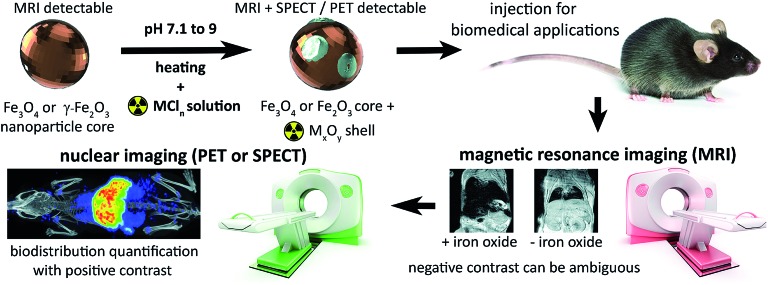
Synthesis of radiolabelled IONPs using radiometal chloride salts (MCl_*n*_) to form an oxidised radiometal coating allows whole-body non-invasive quantitative imaging using PET or SPECT, in addition to high-resolution detection using MRI.

The tuneable properties of iron oxide nanoparticles (IONPs), primarily magnetite, maghemite, or mixtures thereof, and their capacity for functionalisation suit them for numerous biomedical applications: hyperthermia therapy for cancer; drug, cell and gene delivery, and as diagnostic or cell tracking agents.^1^–^7^ The ability of superparamagnetic nanoparticles to de-phase the MRI-detectable water proton (^1^H) signal enables their detection in tissues as hypo-intensities, confirming delivery and retention with high resolution and sensitivity. Yet, in practice, identification of IONPs with magnetic resonance imaging (MRI) is often ambiguous due to endogenous signal hypo-intensities including the lungs, bone, and gut (Fig. 1). Particle quantification is also hampered by saturation of signal loss at high concentrations, insufficient sensitivity to low concentrations, and aggregation effects on relaxivity.^8^,^9^

These limitations have spurred research into conjugation and chelation chemistry for radiolabelling IONPs, to enable their detection and quantification with nuclear imaging.^2^,^10^,^11^ Single photon emission computed tomography (SPECT), and positron emission tomography (PET) detect γ-radiation respectively produced directly or indirectly (after positron annihilation) following radioisotope decay. This offers accurate and sensitive quantification of imaging isotopes across the body, without endogenous background signals from tissue. Traditionally, nanoparticle radiolabelling requires surface functionalisation with organic chelators^10^ – increasing the complexity, time and cost of synthesis. A standard method of radiolabelling IONPs has proven elusive as radiometals differ in co-ordination numbers and atomic radii, therefore requiring different chelating agents and conjugation strategies.^12^

Radiochemical doping provides one alternative to the use of chelators, whereby radiometals such as ^64^Cu and ^111^In are incorporated in the iron oxide core during its synthesis.^13^,^14^ This has the advantage of stable radiolabel retention. However its practicality is reduced by the necessity of synthesising (and possibly functionalising) particles on-demand before every use due to the constraints of isotope half-lives. A more user friendly and clinically-translatable approach would allow last-minute labelling of off-the-shelf iron oxide nanoparticles with the chosen isotope prior to injection. With this in mind, Chen *et al.* demonstrated a post-synthesis, chelate-free method for radiolabelling uncoated iron oxides using radioarsenic (^71^As, ^72^As, ^74^As, ^76^As),^15^ which was followed by a similar demonstration using ^69^Ge, also for PET imaging.^16^ To expand upon this, Boros *et al.* labelled the FDA-approved, carbohydrate coated IONP ferumoxytol (Feraheme®) with a range of more commonly available radiometal isotopes, including ^89^Zr and ^64^Cu for PET and ^111^In for SPECT.^17^ Particles were heated in an aqueous solution with the radiometal chloride (≥80 °C was optimal for most metals tested, including Zr, Cu, and In) at a pH between 7–9. However, despite ongoing interest in this method,^2^,^7^ the nature of the chemical interaction between the metal isotopes and iron oxide nanoparticles remained unidentified.^18^

Here, we address this problem by applying a combination of energy-dispersive X-ray spectroscopy (EDS), X-ray photoelectron spectroscopy (XPS), time of flight mass spectrometry (ToF-SIMS) and room temperature ^57^Fe Mössbauer spectroscopy, to elucidate the mechanistics of heat-induced iron oxide and ferrite radiolabelling as described by Boros *et al.* We establish that it operates primarily through mineralisation of the radiometal onto the particle surface as a radiometal oxide (see Fig. 1). We show that this surface radiomineralisation (SRM) has no effect on the structural and chemical properties of commercially available maghemite and magnetite/maghemite-based IONPs, and that as the key magnetic properties of the particles remain unchanged, their utility for MRI and other biomedical applications is retained. Finally, we demonstrate tracking of ^111^In radiolabelled IONPs using whole-body, non-invasive SPECT imaging, thereby illustrating key advantages over the use of MRI alone.

## Results

We initially investigated the ability of the reported method^17^ to induce radiolabelling of commercially-available nanoparticles representing the two magnetic materials most commonly used in biomedical applications: maghemite (γ-Fe_2_O_3_), and magnetite (Fe_3_O_4_).^4^ As we have shown previously, commercially available magnetite is typically a magnetite/maghemite mixture despite being labelled as >98% magnetite, which we confirmed here using a model-independent fitting of the ^57^Fe Mössbauer spectrum of the “magnetite” sample, as per Fock and Bogart *et al.,*^19^ which indicated a mixture comprising ≈63 wt% magnetite and 37 wt% maghemite. The maghemite and the magnetite/maghemite particles were then labelled *via* heating to 90 °C at pH 9 in the presence of 100 to 200 kBq of either ^111^In or ^89^Zr, for 90 min (Table 1). This resulted in 79% to 94% retention of the radioactivity on the particles; assessed by either thin layer chromatography (TLC) or magnetic separation.

**Table 1:** Radiochemical yield (RCY) independently measured with thin layer chromatography (TLC) and magnetic separation. Correlation between the two measurements across the maghemite and (nominally) magnetite samples was *R*^2^ = 0.93. Reactions without magnetic particles showed negligible activity retention following either TLC or attempted magnetic separation

Nominal chemical composition	Nominal particle diameter	Supplier	Matrix	^111^In RCY (%)		^89^Zr RCY (%)	
				TLC, *n* = 11 (SEM)	Magnetic separation, *n* = 4 (SEM)	TLC, *n* = 10 (SEM)	Magnetic separation, *n* = 4 (SEM)
N/A control	—	—	—	0.7 (0.5)	0.4 (0.2)	2.46 (2.9)	0.4 (0.2)
Maghemite (γ-Fe_2_O_3_)	20–40 nm	Alfa Aesar	Bare	79.1 (4.9)	79.3 (6.8)	94.2 (0.7)	94.7 (0.4)
Magnetite (Fe_3_O_4_)	50–100 nm	Sigma Aldrich	Bare	85.2 (3.1)	78.6 (6.4)	94.9 (1.1)	94.2 (0.7)
Y_3_Fe_5_O_12_	<100 nm	Sigma Aldrich	Bare	88.2 (3.7)	66.6 (3.5)^*a*^	91.9 (1.3)	71.9 (3.5)^*a*^

^*a*^Magnetic separation of Y_3_Fe_5_O_12_ was not completely successful as indicated by visual inspection.

The radiolabelling efficiency was slightly higher using ^89^Zr than with ^111^In, consistent with the previous report on ferumoxytol.^17^ Labelling was repeated using non-radioactive (natural abundance) metal isotopes of InCl_3_ and ZrCl_4_ (10 μmol metal chloride additive per 100 mg of IONPs). Metal additives were successfully incorporated into IONPs as assessed by inductively coupled plasma mass spectrometry, ICP-MS (17 to 51%), giving the same trend of higher labelling efficiencies for Zr compared to In (Table S1†). To show the broader applicability of this process with alternative iron oxides, yttrium iron oxide nanoparticles (Y_3_Fe_5_O_12_) were also successfully radiolabelled (Table 1).

**Fig. 2:**
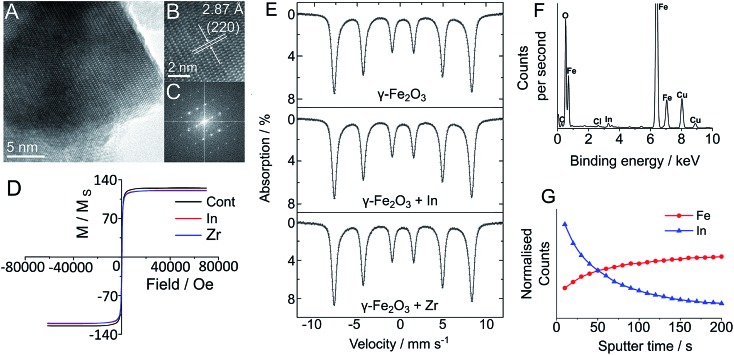
Characterisation of maghemite (Alfa Aesar) γ-Fe_2_O_3_ following ^111^In labelling. High resolution TEM images (A–C), show no discernible change in lattice structure from bulk iron oxide. (C) Lattice *d*-spacing of 2.87 Å assigned as the (220) plane of maghemite. (D) SQUID measurement of maghemite (γ-Fe_2_O_3_) before and after modification shows comparable magnetisation curves. (E) Mössbauer spectra acquired for maghemite (Fe_2_O_3_) particles before and after heat induced labelling with non-radioactive In and Zr additives. No change in the proportion of Fe atoms in a maghemite environment was seen following the labelling reaction. (F) EDS spectrum shows the presence of added In. (G) ToF-SIMS demonstrates a sharp decrease in In concentration with increasing sputter time, indicating its surface bound nature.

In order to establish the effect of In and Zr labelling on the physical properties of magnetite/maghemite and Y_3_Fe_5_O_12_ nanoparticles, we have used both transmission electron microscopy (TEM) and powder X-ray diffraction (pXRD) and observed no discernible change in the physical structure of the nanoparticles following the labelling treatment (Fig. 2). TEM imaging clearly showed that there was no change in the physical structure of the nanoparticles followed the labelling treatment (Fig. 2). The core sizes of each sample displayed the familiar log-normal size distribution, which remained unchanged regardless of reaction conditions. Indeed, due to the highly polydisperse nature of the nanoparticles (as seen by the large standard deviation), any change in size was impossible to see: 35.3 ± 20.5 nm for the maghemite, 19.1 ± 15.8 nm for the Y_3_Fe_5_O_12_ and 116.6 ± 79.0 nm for the magnetite/maghemite. High resolution TEM analysis (Fig. 2A–C and S14–S22†) clearly shows the lack of core/shell structure and no change in the lattice planes of the nanoparticles, consistent with pXRD observations (Fig. S1–S4†). The inability to detect either In or Zr *via* pXRD suggests an amorphous and non-crystalline incorporation, as would be expected based on the relatively low reaction temperature. The presence of small amounts of In and Zr within the samples was confirmed with energy dispersive X-ray spectroscopy (EDS) (Fig. 2F and S14–S22†).

To quantify any change in composition following heating and radiolabelling, the maghemite and magnetite/maghemite particles were analysed with room temperature ^57^Fe Mössbauer spectroscopy^19^ before and after In and Zr labelling (Fig. S23†). For the labelled maghemite samples there was no change in the measured spectra for all of the treated samples; we confirmed this quantitatively by observing no change to the value of the α parameter (the numerical proportion of Fe atoms in the magnetite environment), with *α* = 0 ± 0.04, consistent with pure maghemite.^19^,^20^ Similarly, for the treated Sigma Aldrich “magnetite” samples there was no change to the spectra, with the best fit isomer shift indicating *α* = 0.52 ± 0.02, corresponding to a magnetite content of *ca.* 56 wt%. Such observations strongly suggest that there is no incorporation of either the In or Zr within the sub-lattice structures of the iron oxide, indicating a surface location.

We confirmed this using X-ray photoelectron spectroscopy (XPS; a surface (<10 nm) sensitive technique) to probe the oxidation state and quantities of the In and Zr additives (Fig. S5–S13†). All samples showed the presence and absence of the In and/or Zr additives where appropriate. Fe environments were largely unchanged after In/Zr treatment, with only 0.1 eV variation across the three samples in Fe 2p scans, in good agreement with the Mössbauer spectra. The Fe 2p_3/2_ values of 710.6 (AA maghemite), 710.7 (SA magnetite), and 710.3 eV (Y_3_Fe_5_O_12_) are indicative of γ-Fe_2_O_3_ (710.6, 710.7 eV) and Y_3_Fe_5_O_12_ (710.3 eV) respectively.^21^,^22^ There was also little variation within the Y_3_Fe_5_O_12_ samples, with a single environment, and a variation in the Y 3d_5/2_ range of 157.0–157.3 eV displayed, similar to that observed in Y_2_O_3_.^23^

Both the In and Zr additives were clearly seen in single chemical environments, for all treated samples. Scans of In 3d showed In with a 3+ oxidation state, assigned as In_2_O_3_ at 444.3 eV and Zr 3d scans showed a Zr 3d_5/2_ peak at 182.0 eV assigned as ZrO_2_, with Zr in the 4+ oxidation state. The high oxidation states of the additive elements and the absence of any observable change or indeed new chemical environments in either the O 1s or Fe 2p high resolution scans, leads us to conclude that the additives are surface bound and not fully integrated (doped) into the iron oxide structure, which is supported by pXRD and Mössbauer spectroscopy (Fig. S1–S4†). Based on the amount of In detected in the samples by ICP-MS, this gives a ratio of 1 In atom for every 3 to 20 surface Fe atoms per nanoparticle for the magnetite/maghemite and maghemite particles respectively – consistent with the absence of shell detection with TEM.

The surface location of additives was further established with time of flight-secondary ion mass spectroscopy (ToF-SIMS), which was used to remove atomic monolayers of metal ions from the surface of the IONPs. Fig. 2G presents the ToF-SIMS depth profile for the In doped maghemite (γ-Fe_2_O_3_-In), in which the In concentration shows a sharp drop with etching time. The slight increase in iron concentration can be attributed to its dominance within the core of the NPs, which becomes more clear on removal of the In. These measurements were complemented by the ToF-SIMS depth profile measurements of the Y_3_Fe_5_O_12_ NPs (Fig. S28, ESI†). From this, we propose that the In atoms are surface bound, consistent with the mild nature of the radiolabelling reaction and its negligible effects on the structural (by TEM) and physical (*vide infra*) properties of the particles. ToF-SIMS of the Zr-doped maghemite and Y_3_Fe_5_O_12_ NPs was complicated by the ionisation efficiency of Zr and the overlapping mass with the Y fragment. Furthermore, ToF-SIMS is more surface sensitive than XPS; which means the ion beam penetration was considerably lower than that of the XPS measurements (sampling depth is considerably less).^24^ Despite this, Zr was successfully detected using XPS (Fig. S5–S13†).

Following the demonstration of In and Zr surface mineralisation using this method, we next sought to demonstrate wider utility with a range of maghemite and magnetite-based nano- and microparticles coated for biomedical application. A small selection of commercially-available magnetic nanoparticles was chosen for variation in size and coating, and labelled with either ^111^In or ^89^Zr according to the protocol described above. As with the uncoated maghemite and magnetite/maghemite particles (Table 1), this resulted in efficient radiochemical yields (RCYs) between 68 and 95% as assessed with TLC and magnetic separation (Table 2).

**Table 2:** Radiochemical yield of labelled particles following heating with ^111^In or ^89^Zr at 90 °C for 90 min. Labelling efficiency was assessed using TLC and independently with magnetic separation. Magnetic separation of 50 nm FluidMag was unsuccessful

Particle type	Nominal particle diameter	Matrix	^111^In % RCY		^89^Zr % RCY	
			TLC (SEM), *n* = 11	Magnetic separation (SEM), *n* = 4	TLC (SEM), *n* = 10	Magnetic separation (SEM), *n* = 8
FluidMag	50 nm	Citrate	68.5 (3.1)	—	86.3 (1.8)	—
FluidMag	100 nm	Citrate	70.3 (3.8)	81.1 (7.7)	87.9 (2.2)	83.4 (5.9)
FluidMag	200 nm	Citrate	69.1 (3.5)	84.9 (6.7)	93.3 (0.9)	94.8 (0.5)
Biomag Maxi	3–12 μm	Carboxyl functionalised alkoxysilane	71.4 (3.1)	86.1 (6.0)	82.8 (1.6)	93.3 (1.5)
SiMag	500 nm	Silanol	80.4 (3.1)	83.7 (1.5)	71.5 (2.8)	64.3 (5.0)

To monitor the effects of the radiolabelling procedure on the magnetic properties of a selection of these particles, super-conducting quantum interference device (SQUID) measurements were taken after labelling with non-radioactive ZrCl_4_ and InCl_3_ additives at a ratio 10 μmol per 100 mg particle (see Table S2, Fig. S24–S27†). For each particle type except the Y_3_Fe_5_O_12_ (which showed altered coercivity but not saturation magnetisation or remanence), magnetisation curves were comparable between unmodified particles and those labelled with Zr and In (Fig. 2D).

**Fig. 3:**
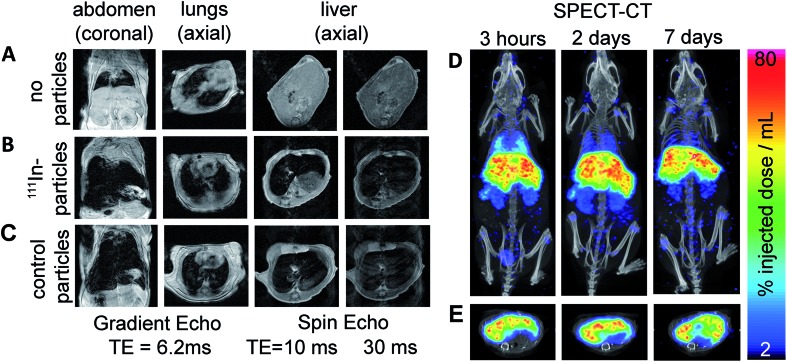
MRI pre (A) and post (B) injection of the ^111^In-FluidMagCT, or (C) post-FluidMagCT (control). (D) Maximum intensity projection ^111^In SPECT-CT at 3 h, 2 and 7 d post-injection confirms presence of labelled iron oxides in the liver, lung, kidneys, and spleen of C57BL/6 mice. (E) Corresponding axial slices show co-localisation of the radiolabelled IONPs and the liver.

We lastly demonstrate that particles labelled with this method are suitable for *in vivo* imaging with MRI and SPECT. FluidMag CT was chosen as a representative particle for biomedical application as it is commercially available and has previously been evaluated for stem cell labelling and hyperthermia.^25^,^26^ MRI was done prior to and 2.5 h following intravenous injection to monitor the distribution of the labelled particles in wild type mice (C57BL/6 strain). Signal hypo-intensity was present in the lungs only pre-injection (Fig. 3A), and post-injection in lungs, liver, and kidneys (Fig. 3B) – consistent with previous reports of excretory organ nanoparticle uptake.^27^ An equivalent dose of unmodified stock particles were injected into a separate animal, and gave comparable distribution of MRI contrast as the radiolabelled particles, predominantly in the liver (Fig. 3B and C).

SPECT-CT imaging at 3 h, 2 d, and 7 d post injection confirmed the location of the ^111^In-labelled IONPs within the liver and kidneys (see Fig. 3), showing additional retention within the lungs which was difficult to identify on the MR images due to endogenous contrast. At 2 and 7 d SPECT-CT showed a clearing of the particles from the lungs – again not detectable using MRI. Quantification of total activity with SPECT ROI analysis showed the majority of activity (54.5%) retained in the liver after 7 days.

## Conclusion

In conclusion, using a combination of TEM, ^57^Fe Mössbauer spectroscopy, XPS, ToF-SIMS and SQUID we have demonstrated that heat-mediated chelate-free radiolabelling method as described by Boros *et al.*^17^ operates by mineralisation of the radiometal (^111^In or ^89^Zr) as an oxide on the surface of the IONP that does not alter the magnetic and physical properties of the particle core. Further, we have shown that this surface radio-mineralisation (SRM) is compatible with a range of nano- and microscale iron oxide particles (maghemite and magnetite/maghemite), as well as yttrium iron oxide (Y_3_Fe_5_O_12_) as a representative non-typical iron oxide. ToF-SIMS analysis shows radiometal incorporation is limited to the surface of the particles, consistent with the lack of change in the magnetite–maghemite content of the starting material. With the exception of a slight effect on the Y_3_Fe_5_O_12_ particles this was confirmed by means of SQUID analysis, which showed that their original magnetisation curves were retained following labelling.

This report affords a better understanding of the heat-induced chelate-free radiolabelling method. We anticipate that this will encourage its use in investigating the bio-distribution of the IONP-based biomedical therapeutics and diagnostics, thus combining the quantitative high-sensitivity of PET/SPECT imaging with the high-resolution detail of MRI.

## Experimental section

Detailed descriptions of the instrumentation, materials and experimental protocols can be found in the ESI.†

## Ethical statement

All animal studies were approved by the University College London Biological Services Ethical Review Committee and licensed under the UK Home Office regulations and the Guidance for the Operation of Animals (Scientific Procedures) Act 1986 (Home Office, London, United Kingdom). All animal methods were performed in accordance to institutional ethical guidelines and regulations. During all *in vivo* imaging, mice were maintained at 37 °C under isofluorane breathable anaesthesia (1–2%) in oxygen. A small animal physiological monitoring system (SA Instruments, Stony Brook, NY) was used to maintain respiration rate. Mice (C57BL/6; male) were obtained from Charles River at 4 months old.

## Conflicts of interest

There are no conflicts to declare.

## Supplementary Material

Supplementary information
