# Supplementary material for: Surface radio-mineralisation mediates chelate-free radiolabelling of iron oxide nanoparticles
Source: Chem Sci. 2019 Jan 9;10(9):2592–7. doi: 10.1039/c8sc04895a (PMC6419938; doi:10.1039/c8sc04895a)
Supplement: Supplementary file 1 [file SC-010-C8SC04895A-s001.pdf]

Electronic Supplementary Material (ESI) for Chemical Science.
This journal is © The Royal Society of Chemistry 2019

Surface Radio-Mineralisation Mediates Chelate-free Radiolabelling of Iron Oxide Nanoparticles

P. Stephen Patrick*, Lara K. Bogart, Thomas J. Macdonald, Paul Southern, Michael J. Powell, May Zaw-Thin, Nicolas H. Voelcker, Ivan P. Parkin, Quentin A. Pankhurst, Mark F. Lythgoe, Tammy L. Kalber, and Joseph C. Bear*

P. Stephen Patrick, May Zaw-Thin, Mark Lythgoe, Tammy Kalber- Centre for Advanced Biomedical Imaging (CABI), Department of Medicine, University College London, London WC1E 6DD,
**UK. Email: peter.patrick@ucl.ac.uk; j.bear@kingston.ac.uk*

Joseph C. Bear, School of Life Science, Pharmacy & Chemistry, Kingston University, Penrhyn Road, Kingston upon Thames, UK, KT1 2EE.

Thomas J. MacDonald, Michael J. Powell, Ivan P. Parkin- Materials Chemistry Centre, Department of Chemistry, University College London, 20 Gordon Street, London, WC1H 0AJ, UK.

Lara K. Bogart, Paul Southern, Quentin A. Pankhurst- UCL Healthcare Biomagnetics Laboratories, 21 Albemarle Street, London, W1S 4BS, UK.

Nicolas H. Voelcker –(1) Monash Institute of Pharmaceutical Sciences, Monash University, Parkville, Australia. (2) Commonwealth Scientific and Industrial Research Organisation (CSIRO), Clayton, Australia

Materials:

$InCl_3$ (anhydrous powder >98% purity) and $ZrCl_4$ (anhydrous powder, >99.9% trace metal basis) were obtained from Sigma Aldrich. NaOH (1 N) was prepared using NaOH pellets (>98%, BioUltra, Sigma Aldrich), and distilled water (Milli-Q, 18.2 MΩ•cm).
HEPES buffer (0.2M, pH 7) was prepared using HEPES (4-(2-Hydroxyethyl)piperazine-1-ethanesulfonic acid, *N*-(2-Hydroxyethyl)piperazine-N-(2-ethanesulfonic acid)) powder (>99.5% titration; Sigma Aldrich), dissolved in distilled water (Milli-Q, 18.2 MΩ•cm).

Nanoparticles were obtained from several sources: $Y_3Fe_5O_{12}$ (Sigma Aldrich Ltd.), Maghemite (NanoArc® iron oxide, Alfa Aesar, 20 to 40 nm), Magnetite (Iron(II,III) oxide, 50 to 100nm, Sigma Aldrich, 637106), FluidMag CT in 50 nm, 100 nm and 200 nm sizes (Chemicell, 25 mg/mL), Biomag® Maxi Carboxyl 3-12 μm, carboxyl coated (Bangs Laboratories Inc., 20 mg/mL), SiMag Silanol 0.5 μm (Chemicell, 50 mg/mL).

^{89}Zirconium was obtained from Perkin Elmer dissolved in 1M Oxalic acid. $^{111}InCl_3$ was obtained from Mallinckrodt, supplied in 1 M HCl.

Methods

Bulk sample preparation of iron oxide particles labelled with non-radioactive metal chlorides:

100 mg of Iron oxide nanoparticles (20 to 40 nm, Fe_2O_3, maghemite, NanoArc®, Alfa Aesar), (50 to 100 nm, Fe_3O_4 magnetite, Sigma Aldrich) or Yttrium Iron Oxide nanoparticles (< 100 nm, $Y_3Fe_5O_{12}$, Sigma Aldrich Ltd.) were diluted with 40 mL of water, to which was added 5 mL of a 2 mM solution of either $InCl_3$ or $ZrCl_4$, or without metal chloride as a control. This was heated to 90˚C for 90 minutes using a water bath, after the addition of 200 µL of NaOH (1M). The reaction was neutralised using 4 mL of HEPES buffer (pH 7, 200 mM), and unbound In and Zr were chelated with DTPA solution (4 mL, 50mM, pH 7.0). Particles were then purified and washed three times using DTPA solution to remove any unbound metal ions.

Radio-labelling and analysis:

Iron oxides were suspended at 10 mg/mL in HPLC grade water to a final volume of 100 µL. $^{111}InCl_3$ or ^{89}Zr oxalate in 1M HCl was added in a volume of 1 µL, followed by 2.5 µL of NaOH (1 M). Samples were then vortexed, and heated at 90˚C for 90 minutes. After cooling to room temperature, 10 µL HEPES buffer (pH 7, 200 mM) was added to neutralise, and 10 µL DTPA solution (50 mM; pH 7.5) to bind unreacted indium or zirconium. Samples were incubated at room temperature for a further 90 minutes. Radiochemical purity was then analysed with thin layer chromatography (TLC), using aluminium foil-backed silica gel matrix strips (1 cm x 10 cm x 200 µm; Sigma Aldrich) as the stationary phase. A drop of the sample (2 µL) was loaded at a designated origin and left to air dry, before eluting with freshly-prepared 50 mM DTPA (Diethylenetriaminepentaacetic acid) solution (pH 7.5) as the mobile phase, taking care that the sample origin was not immersed. Free indium and zirconium are chelated by DTPA and move with the solvent front, whereas particle-bound activity remains at the origin. TLC strips were cut at Rf 0.1 and activity from each section quantified using a gamma counter (Wizard2, Perkin Elmer). Radiochemical purity was calculated as the percentage of the total activity from both sections of the strip that was below R_f 0.1. The remaining sample was then purified using a MACSIMAG™ (Miltenyi Biotech) separator to isolate the magnetic particles from the supernatant. After initial separation, particles were washed once with 1 mL DTPA solution (50 mM, pH 7.5), and re-separated. Particle-bound activity and supernatant activity was then quantified using a gamma counter. All particles except FluidMag CT 50 nm, and $Y_3Fe_5O_{12}$ were successfully separated from solution.

Preparation of particles for in vivo imaging:

100 µL of stock solution of a 200 nm diameter citrate-coated iron oxide particle (FluidMag CT, Chemicell, 25 mg/mL), were mixed with 50 µL of $^{111}InCl_3$ solution (30 MBq), and 2 µL of a 1 N Na_2CO_3 solution, adjusting the pH to 8.5. This was sealed in a screwcap Eppendorf tube and heated to 90 degrees for 1 hour. This was then neutralised using 50 µL of HEPES (4-(2-hydroxyethyl)-1-piperazineethanesulfonic acid) buffer (200 mM, pH 7), and 10 µL DPTA (50 mM, pH 7.0) solution, and then purified using a MACSIMAG (Miltenyi Biotech) separator to isolate the magnetic particles from the supernatant. 0.75 mg of particles in 200 µL HEPES buffer solution were then injected intravenously *via* the tail vein of a mouse, and SPECT-CT imaging was performed at 3 h, 48 h, and 7 days after injection.

In vivo imaging:

All animal studies were approved by the University College London Biological Services Ethical Review Committee and licensed under the UK Home Office regulations and the Guidance for the Operation of Animals (Scientific Procedures) Act 1986 (Home Office, London, United Kingdom). All animal methods were performed in accordance to institutional ethical guidelines and regulations. During all *in vivo* imaging, mice were maintained at 37 ºC under isofluorane breathable anaesthesia

(1-2%) in oxygen. A small animal physiological monitoring system (SA Instruments, Stony Brook, NY) was used to maintain respiration rate. Mice (C57BL/6; male) were obtained from Charles River at 4 months old.

Instrumentation:

XPS

X-Ray photoelectron spectroscopy (XPS) was performed using a Thermo Scientific K-alpha spectrometer with monochromated Al Kα radiation, a dual beam charge compensation system and constant pass energy of 50 eV (spot size 400 μm). Survey scans were collected in the range 0 – 1200 eV. High-resolution peaks were used for the principal peaks of C (1s), Fe (2p), In (3d), O (1s), Y (3d) and Zr (3d). Peaks were modelled with CASA XPS software.

XRD

X-ray diffraction (XRD) studies were carried out using a Stoe (Mo) StadiP diffractometer. The instrument operates with a Mo X-ray source (Mo tube 50 kV 30 mA), monochromated (Pre-sample Ge (111) monochromator selects Kα1 only) and a Dectris Mython 1k silicon strip detector covering 18° 2θ. Samples were run in transmission mode, with the sample being rotated in the X-ray beam. The diffraction patterns obtained were compared with database standards.

TEM

Transmission electron microscopy (TEM) images were obtained using a TEM Jeol 2100 with a LaB_6 source operating at an acceleration voltage of 200 kV. Micrographs were recorded on a Gatan Orius Charge-coupled device (CCD). The powders were sonicated in *n*-hexane and drop-cast onto a 400 Cu mesh lacey carbon film grid (Agar Scientific Ltd) for TEM analysis. Energy dispersive X-ray spectra (EDS) were recorded on an Oxford Instruments XMax EDS detector using AZTEC software.

ToF-SIMS

Time-of-flight secondary ion mass spectrometry (ToF-SIMS) was used to perform elemental depth profiles on iron oxide and doped iron oxide powders. The ToF-SIMS instrument was a Physical Electronics TRIFT V NanoToF. Depth profiles were conducted using a liquid metal Au^+ ion gun (LMIG) operated at 30 kV. The ToF-SIMS measurements were carried out using a raster size (total area sputtered) of approximately 200 μM.

SPECT

SPECT data was acquired using a NanoScan SPECT-CT (Mediso), interfaced to a computer running Interview Fusion software (Bartec). Images were reconstructed using HiSPECT software, and analysed using VivoQuant software (inviCRO). CT images were acquired using a 55 kVP X-ray source, 500 ms exposure time, 180 projections, a pitch of 1.5, and a total scan time of 3 minutes 45s. SPECT Images were obtained using a 4-head scanner with nine 1.4 mm pinhole apertures in helical scan mode using a time per projection of 60s resulting in a scan time of 40 minutes. CT images were reconstructed in voxel size 124 x 124 x 124 μm, whereas SPECT images were reconstructed in a 256 × 256 matrix prior to being overlayed. 3D ROIs were manually drawn around the liver, lungs, and kidneys, and used to calculate the percentage of injected dose/organ (%ID/organ).

MRI

Magnetic resonance images were obtained using a 1T Bruker ICON desktop MRI system (Bruker BioSciences Corporation, Ettlingen, Germany), interfaced to an operating console running Paravision 5 software (Bruker). A 38 mm mouse body solenoid RF coil (Bruker) was operated on transmit/receive mode. Multi-slice images were acquired using a spoiled gradient echo sequence

with 12 slices, TE of 6.2 ms and a TR of 387 ms, 2 averages, and an 80° flip angle. Images were acquired for a 4 × 4 cm field of view with 256 × 256 in plane resolution, and 20 slices of 2 mm thickness with 2.25 mm separation. Total scan time was 3 minutes 19 seconds per animal. T_2-weighted multislice images (5 slices) were acquired axially using a spin echo sequence with 180° refocusing pulse; TEs = 10 to 120 ms in 10 ms increments; TR = 2000 ms. This was done using a field of view of 4 × 4 cm with a 192 x 192 matrix, with slices of 2 mm and inter-slice distance of 3.5 mm. Scan time was 6 minutes 24s.

SQUID Magnetometry

Magnetometry data was acquired using a Quantum Design MPMS-7T SQUID VSM (Quantum Design, USA). Dried powdered samples were loaded into polycarbonate holders and mounted onto the VSM transport rod. MH measurements were performed at 300 K using logarithmically spaced fields between ±7 T.

Mossbauer spectroscopy:

Powdered samples were prepared for measurement by mixing with sucrose to form a solid dispersion, and mounted in a 2.1 cm coin shaped absorber, in line with the standard operating procedure guidelines given in [1].

The spectrometer was operated in transmission geometry and in constant acceleration mode. A ^{57}Co in Rh foil was the source of the 14.4 keV γ-rays, with velocity calibration performed by recording a reference spectrum from a 10 μm thick foil of α-Fe, also at room temperature. All spectra were folded and baseline corrected using a cubic spline correction derived from the α-Fe calibration spectrum and following a protocol implemented in the Windows-based Recoil analysis program [2].

All spectra were fit using the Windows based Recoil curve fitting program (see above reference), by applying the model independent, so called ‘centre of gravity’ method described in [1] to determine the best fit (lowest χ^2) to the spectrum. The ‘centre of gravity’ method allows for the area weighted mean isomer shift of a magnetically split ^{57}Fe Mössbauer spectrum to be determined without the need to assume any specific underlying model for the microenvironment of the Fe atoms, provided that the data is obtained from of thin, texture free absorbers and possesses a flat, well defined background and that the sample comprises magnetite/maghemite only. For all spectra, this was obtained using Voigtian lineshapes (Gaussian distributions of Lorentzian lines). All sub-spectra were constrained to a 3:2:1 area ratio. A value of alpha, the numerical proportion of Fe atoms in the magnetite environment, was obtained from the best fit isomer shift.

Tables:

Particle type	In (labelling efficiency)	Zr (labelling efficiency)	In to Fe ratio	Zr to Fe ratio
Alfa Aesar Maghemite (γ-Fe_2O_3)	17.8 %	51.3 %	1:704	1:244
Sigma Aldrich Magnetite (Fe_3O_4)	36.5 %	38.4 %	1:355	1:338
Sigma Aldrich Yttrium Iron Oxide $Y_3Fe_5O_{12}$	34.4 %	42.4 %	1:108	1:87

Table S1. ICP-MS analysis of labelling efficiency of 10 mg samples of the indicated particles with 10 μmol non-radioactive metal chloride additive ($InCl_3$ or $ZrCl_4$). Numbers show the % of the total amount of metal additive that was recovered following the reaction, washing and magnetic separation of the particles, as assessed using ICP-MS. The lower chemical yield here than in the radiolabelling assay (table 1) may be accounted for by use of the greater amount of metal salt, some of which remained unreacted.

X-ray diffraction:

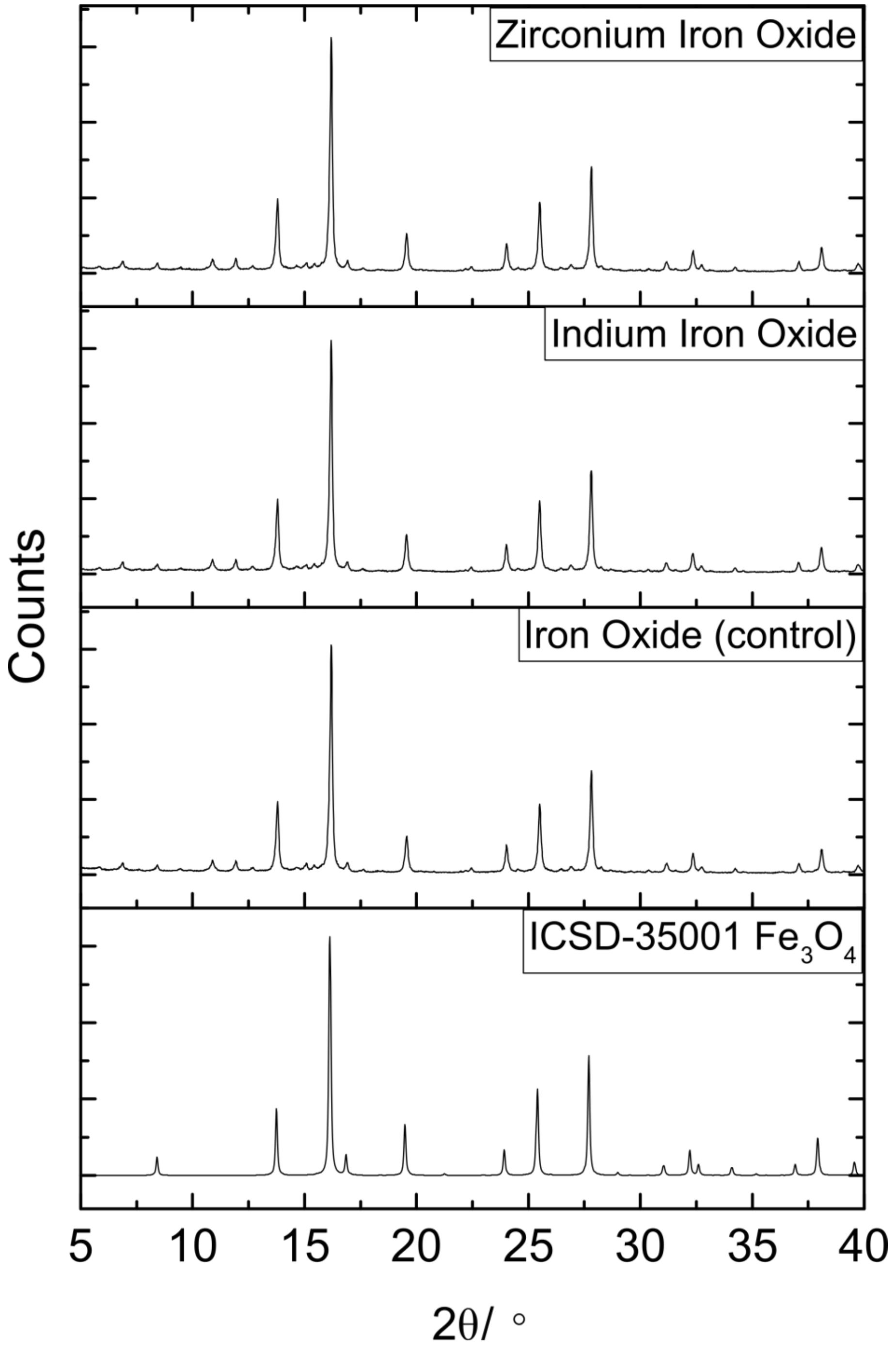

Figure S1: Powder X-ray diffraction (pXRD) patterns of doped and non-doped maghemite (γ-Fe_2O_3) nanoparticles demonstrated no discernible changes in addition of zirconium or indium. This supports surface doping or a limited amount of Fe – Zr or In cation exchange, as a shift in 2θ would indicate doping throughout the structure.

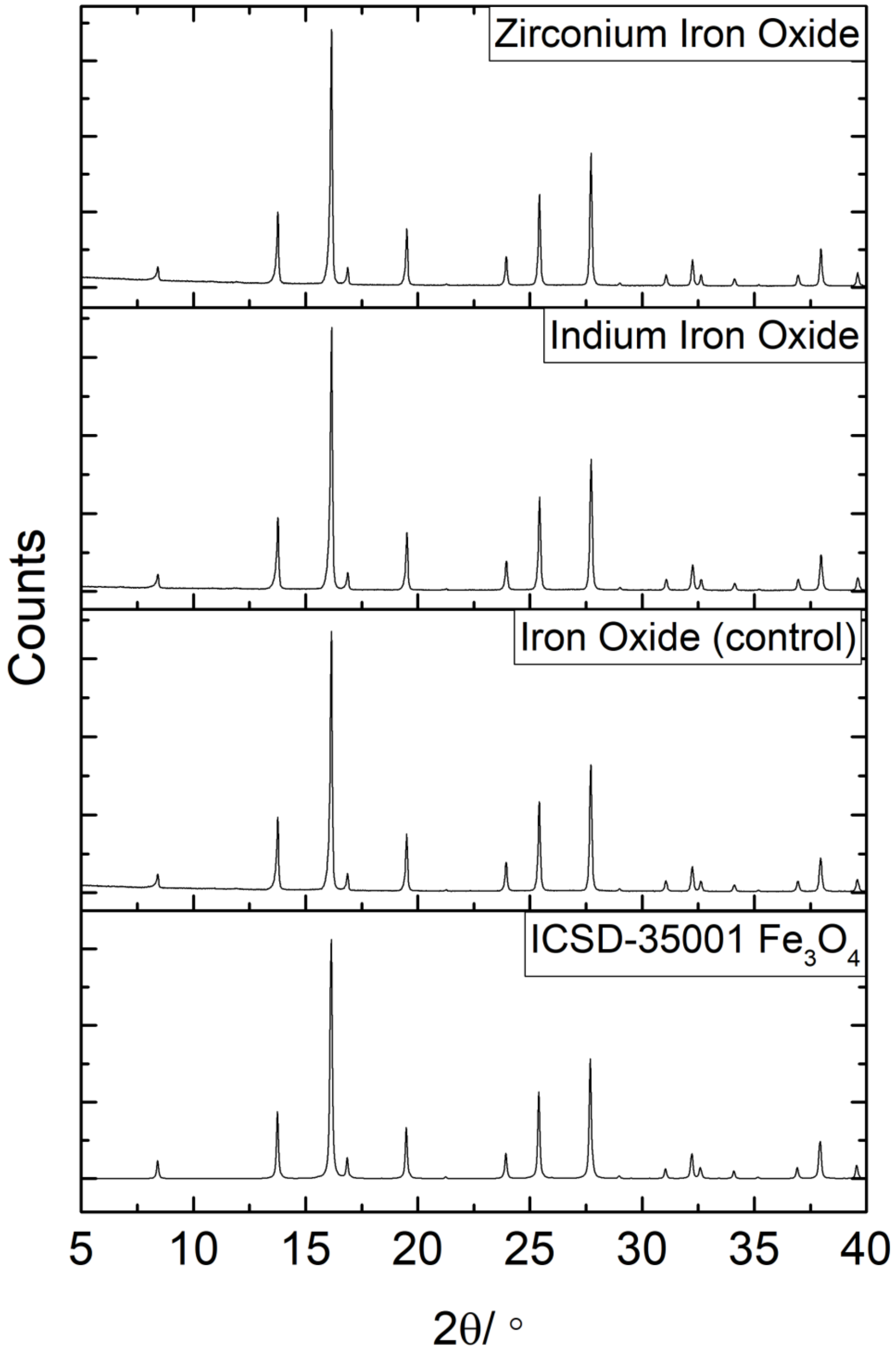

Figure S2. Powder X-ray diffraction (pXRD) patterns of doped and non-doped magnetite (Fe_3O_4) nanoparticles demonstrated no discernible changes in addition of zirconium or indium.

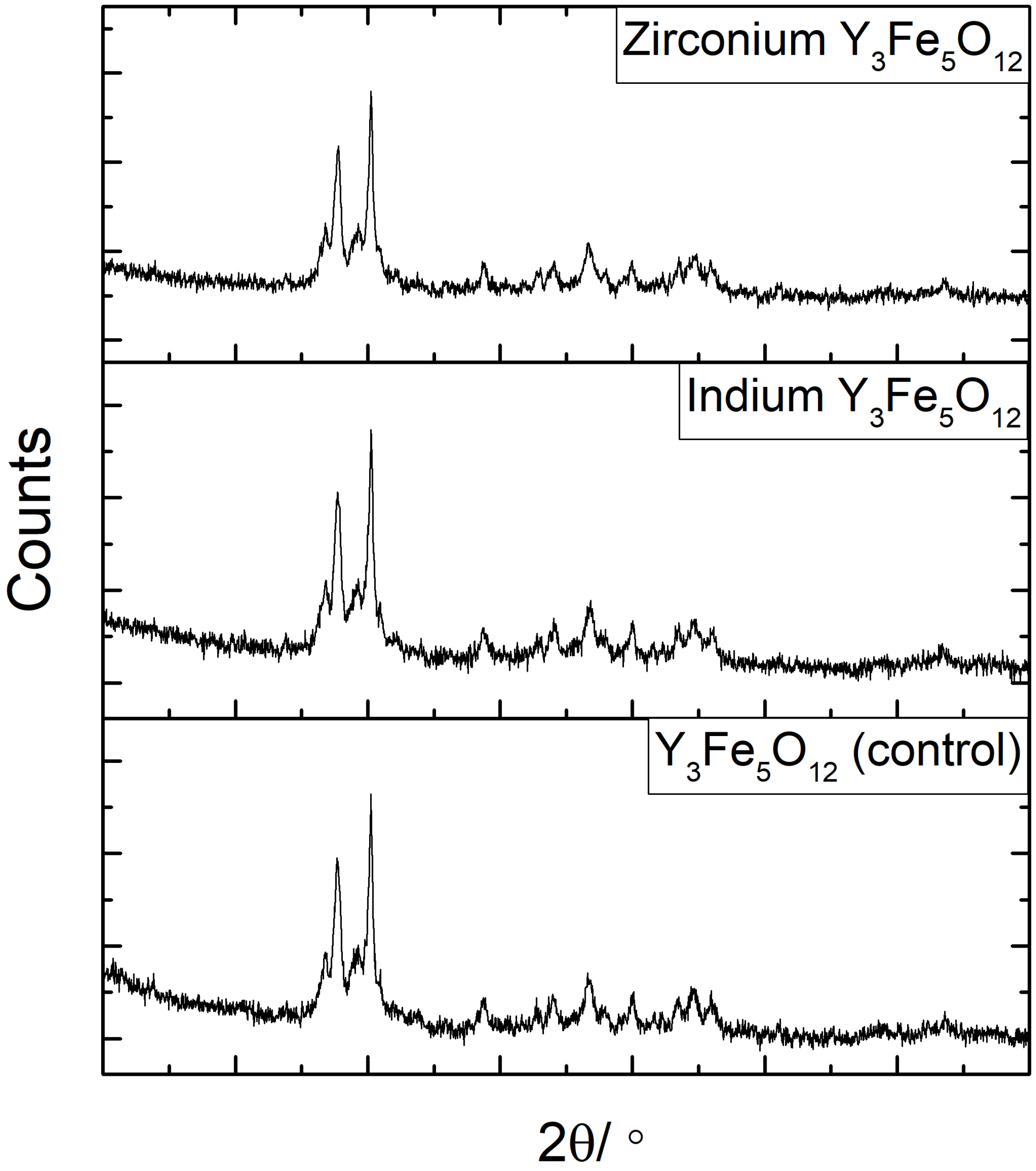

Figure S3. Powder X-ray diffraction (pXRD) patterns of doped and non-doped Yttrium iron oxide ($Y_3Fe_5O_{12}$) nanoparticles demonstrated no discernible changes in addition of zirconium or indium.

(Coupled TwoTheta/Theta)

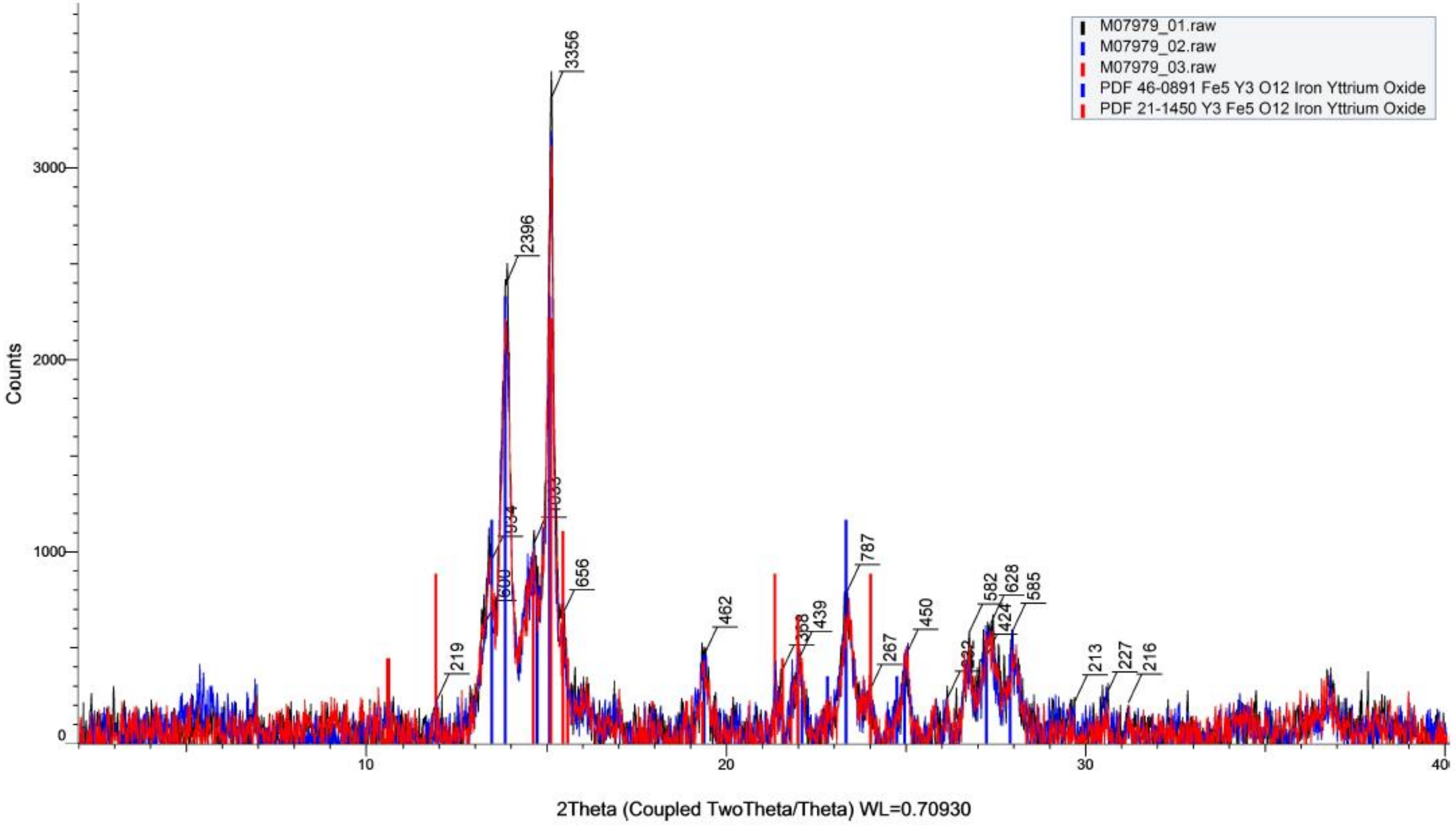

Figure S4. Powder X-ray diffraction (pXRD) reference patterns of doped and non-doped yttrium iron oxide ($Y_3Fe_5O_{12}$), showing a structure match for a mixture of the orthorhombic and tetragonal forms of $Y_3Fe_5O_{12}$. Reference patterns: 46-0891 (*J. Mater. Sci. Lett.* 1990, Vol. 9, page 1314) and 21-1450 (*J. Am. Ceram. Soc.* 1968 Vol. 51, page 713).

X-ray photoelectron spectroscopy:

X-ray photoelectron spectroscopy (XPS) was used to analyse all samples, and was effective at detecting trace elements. In all cases, doublet separation values were taken from the NIST X-ray Photoelectron Spectroscopy (XPS) Database, Version 3.5 and were as follows: In3d, 7.60 eV, Y3d, 2.00 eV and Zr3d 2.40 eV. High resolution spectra were fitted with CASA XPS software and a Gaussian/Laurentzian value of 30. All spectra were calibrated to adventitious carbon at 284.8 eV. A table of peak positions is given at the end of this section, and a detailed explanation of the spectra can be found in the main manuscript. In addition, XPS also showed that all samples exhibited very similar C1s and O1s profiles in their respective groups, with little variation in peak position across both C1s and O1s. Both were fitted with 3 environments, with the C1s assigned as contamination from adventitious carbon: C-C (adventitious carbon, ~ 284.8 eV), C-O-C (adventitious carbon, ~ 286.3 eV) and O-C=O (adventitious carbon, ~288.7 eV). O1s was more sample group specific, with the both the Alfa Aesar and Sigma Aldrich samples having an oxygen environment at ~ 529.8 eV, (magnetite-maghemite), (~531.0 eV for $Y_3Fe_5O_{12}$), C-O and C=O from adventitious carbon at ~531.7 eV and ~533.1 eV respectively.

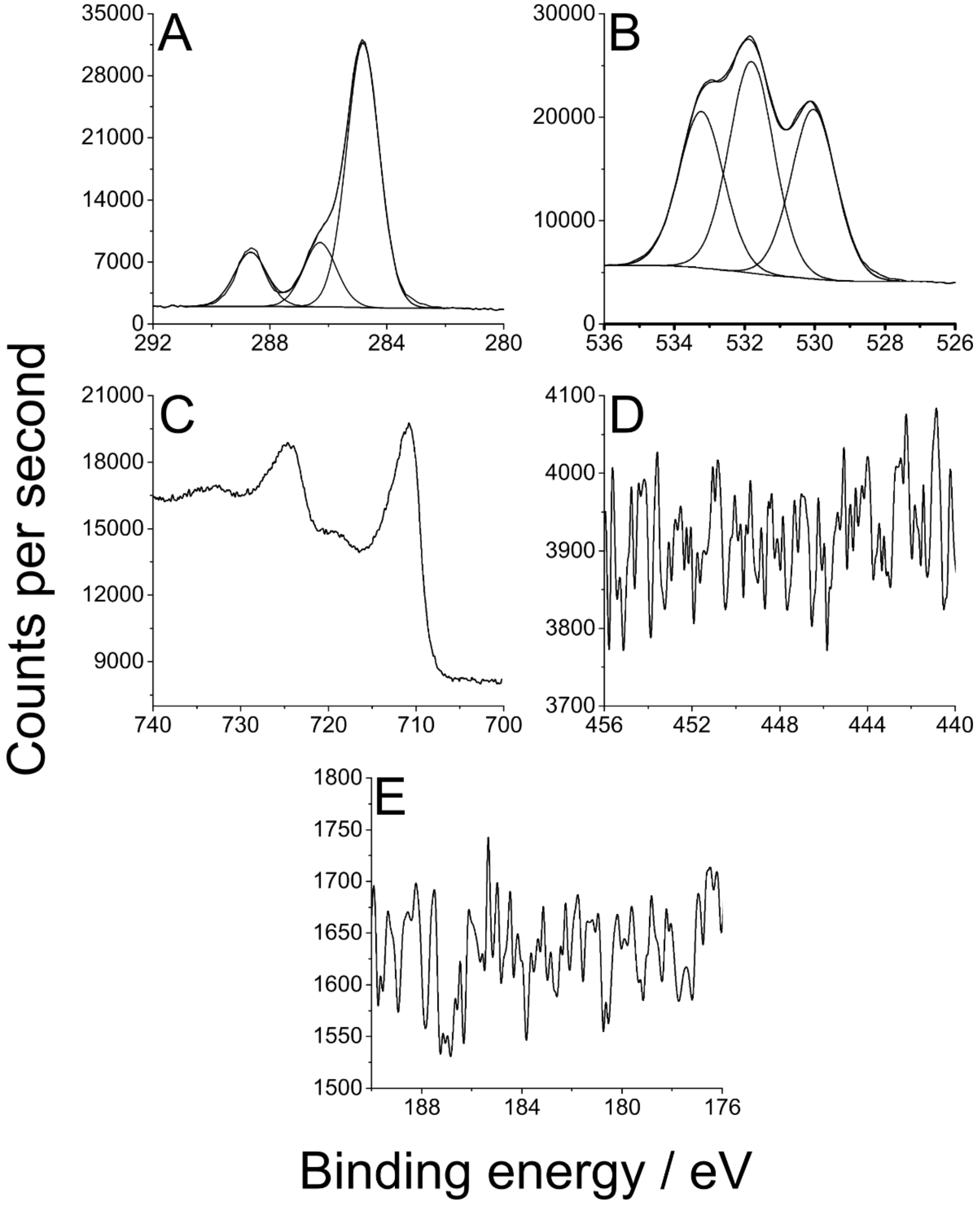

Figure S5: High resolution XPS spectra of the magnetite control sample, showing: A: C1s (3 environments at 284.8 eV, 286.3 eV and 288.7 eV), B, O1s (3 environments at: 530.0 eV, 531.8 eV and 533.2 eV) C: Fe2p (peaks at 710.7 eV ($Fe2p_{3/2}$) and 724.4 eV($Fe2p_{1/2}$)), D: In3d and E: Zr3d. Note the complete absence of In and Zr with no signal seen in spectra D and E.

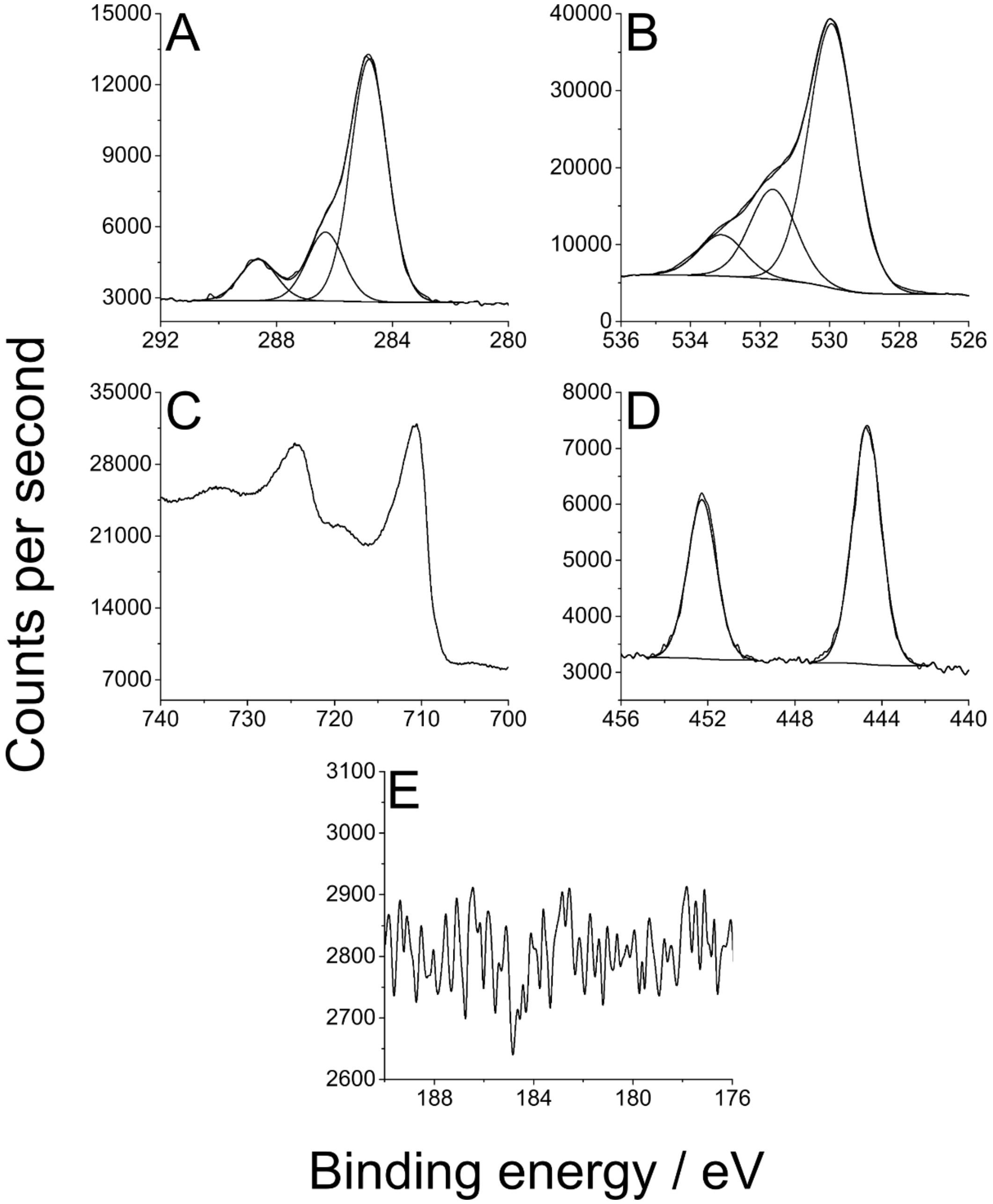

Figure S6: High resolution XPS spectra of the magnetite-indium sample, showing: A: C1s (3 environments at 284.8 eV, 286.3 eV and 288.6 eV), B, O1s (3 environments at: 529.9 eV, 531.6 eV and 533.1 eV), C: Fe2p (peaks at 710.6 eV ($Fe2p_{3/2}$) and 724.3 eV($Fe2p_{1/2}$)), D: In3d (1 environment at 444.7 eV ($In3d_{5/2}$) and 452.3 eV($In3d_{3/2}$)) and E: Zr3d. Note the complete absence of Zr with no signal seen in spectrum E.

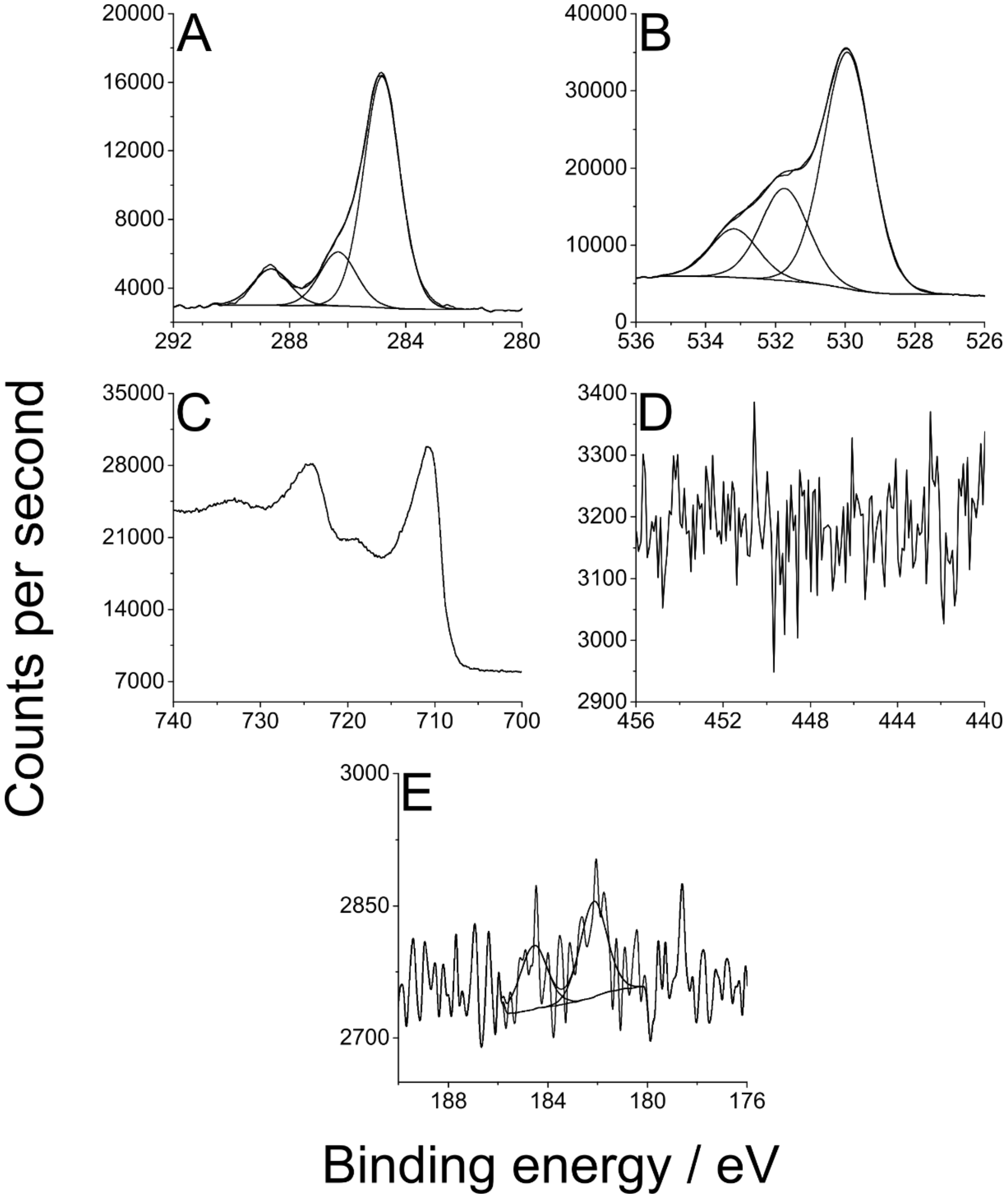

Figure S7: High resolution XPS spectra of the magnetite-Zr sample, showing: A: (3 environments at 284.8 eV, 286.3 eV and 288.6 eV), B, O1s (3 environments at: 529.9 eV, 531.7 eV and 533.2 eV), C: Fe2p (peaks at 710.7 eV (Fe2$p_{3/2}$) and 724.3 eV(Fe2$p_{1/2}$)), D: In3d and E: Zr3d ((1 environment at 182.2 eV (Zr3$d_{5/2}$) and 184.6 eV(Zr3$d_{3/2}$)). Note the complete absence of In with no signal seen in spectrum D.

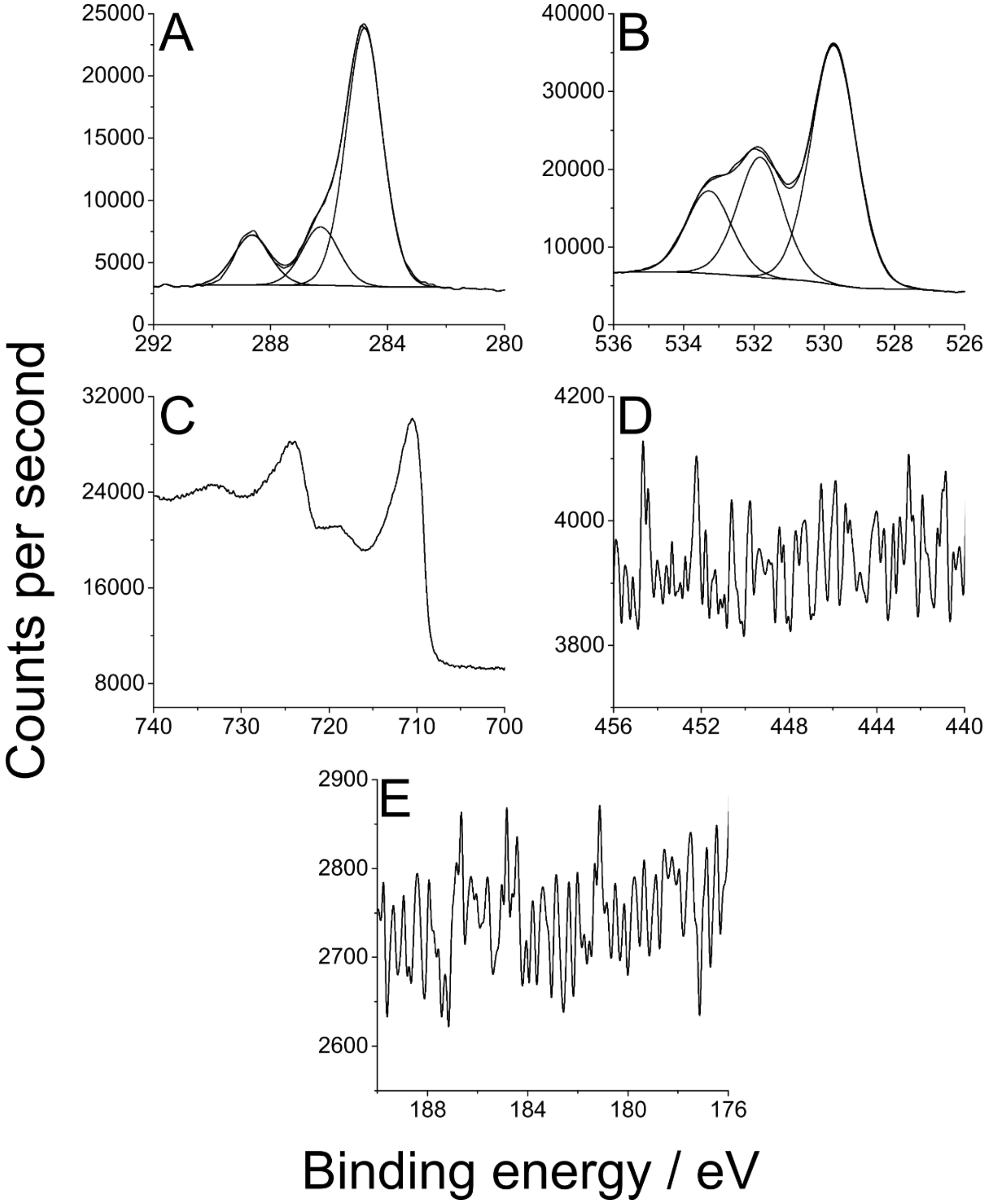

Figure S8: High resolution XPS spectra of the maghemite (Nanoarc ®) control sample, showing: A: C1s (3 environments at 284.8 eV, 286.3 eV and 288.6 eV), B, O1s (3 environments at: 529.7 eV, 531.8 eV and 533.3 eV), C: Fe2p (peaks at 710.6 eV ($Fe2p_{3/2}$) and 724.2 eV($Fe2p_{1/2}$)), D: In3d and E: Zr3d. Note the complete absence of In and Zr with no signal seen in spectra D and E.

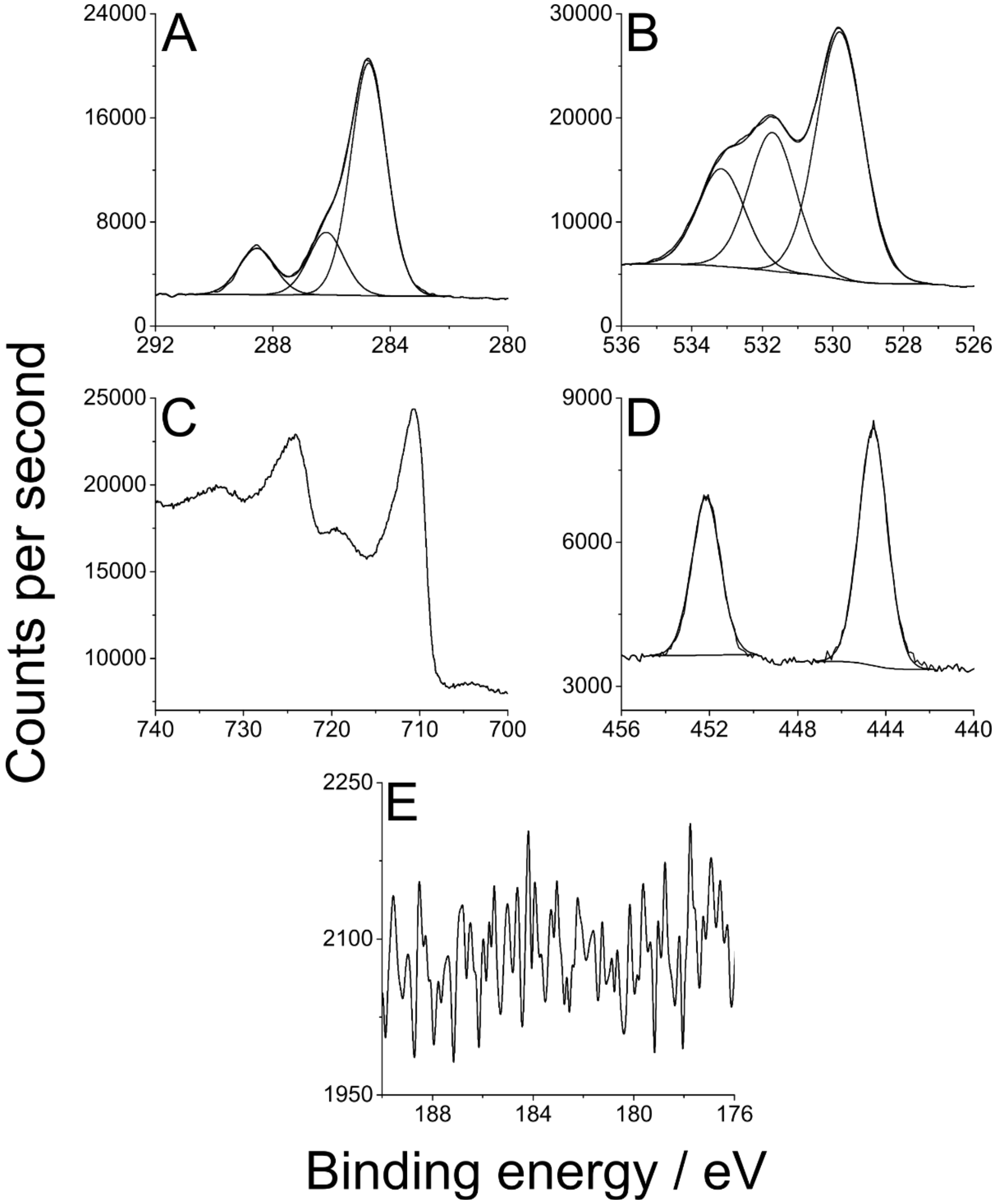

Figure S9: High resolution XPS spectra of the maghemite (Nanoarc ®) In sample, showing: A: C1s (3 environments at 284.7 eV, 286.2 eV and 288.5 eV), B, O1s (3 environments at: 529.8 eV, 531.7 eV and 533.2 eV), C: Fe2p (peaks at 710.7 eV ($Fe2p_{3/2}$) and 724.2 eV($Fe2p_{1/2}$)), D: In3d (1 environment at 444.6 eV ($In3d_{5/2}$) and 452.2 eV($In3d_{3/2}$)) and E: Zr3d. Note the complete absence of Zr with no signal seen in spectrum E.

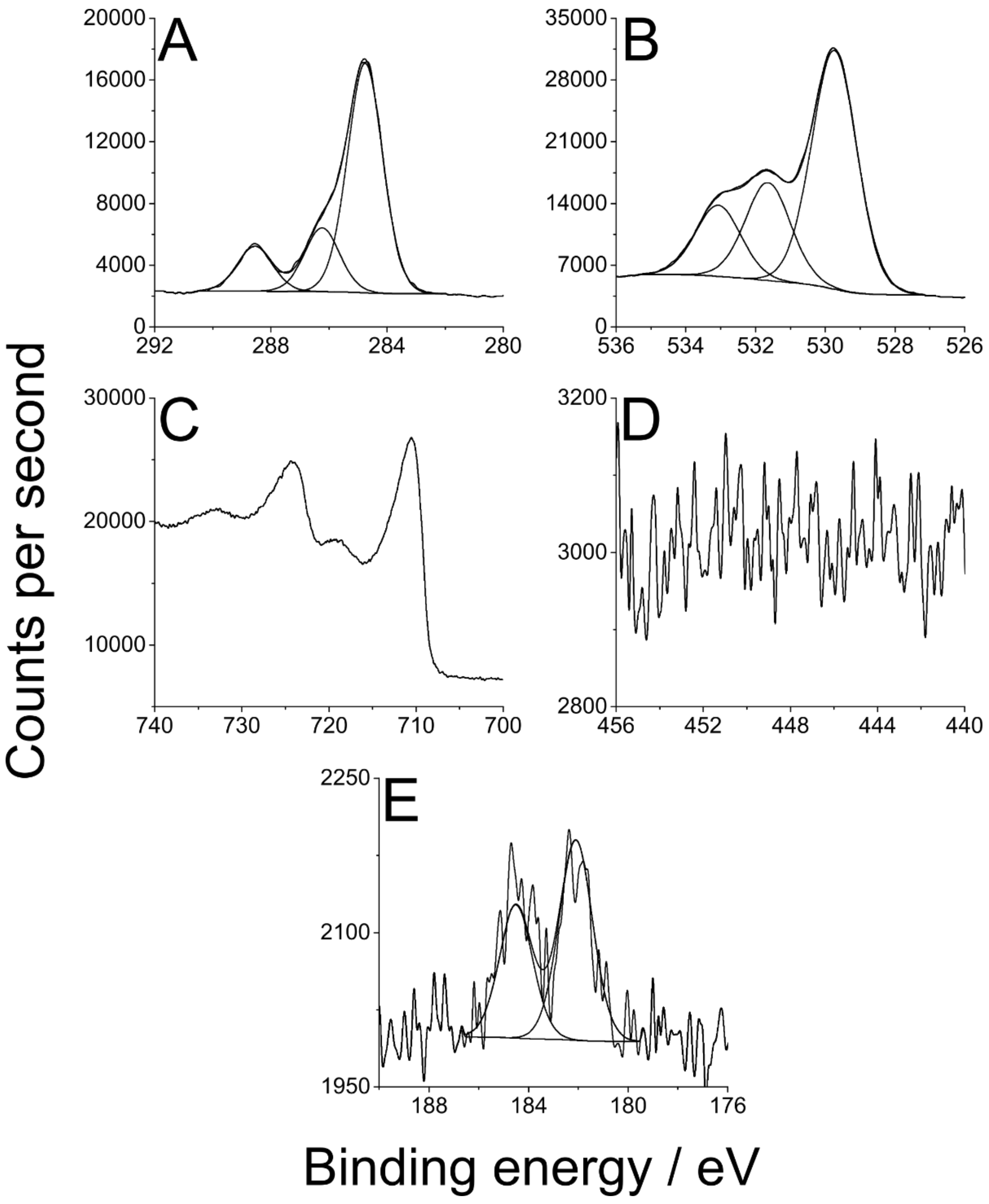

Figure S10: High resolution XPS spectra of the maghemite (Nanoarc ®) Zr sample, showing: A: C1s (3 environments at 284.8 eV, 286.2 eV and 288.6 eV), B, O1s (3 environments at: 529.7 eV, 531.6 eV and 533.1 eV), C: Fe2p (peaks at 710.6 eV ($Fe2p_{3/2}$) and 724.2 eV($Fe2p_{1/2}$)), D: In3d and E: Zr3d (1 environment at 182.1 eV ($Zr3d_{5/2}$) and 184.5 eV($Zr3d_{3/2}$)). Note the complete absence of In with no signal seen in spectrum D.

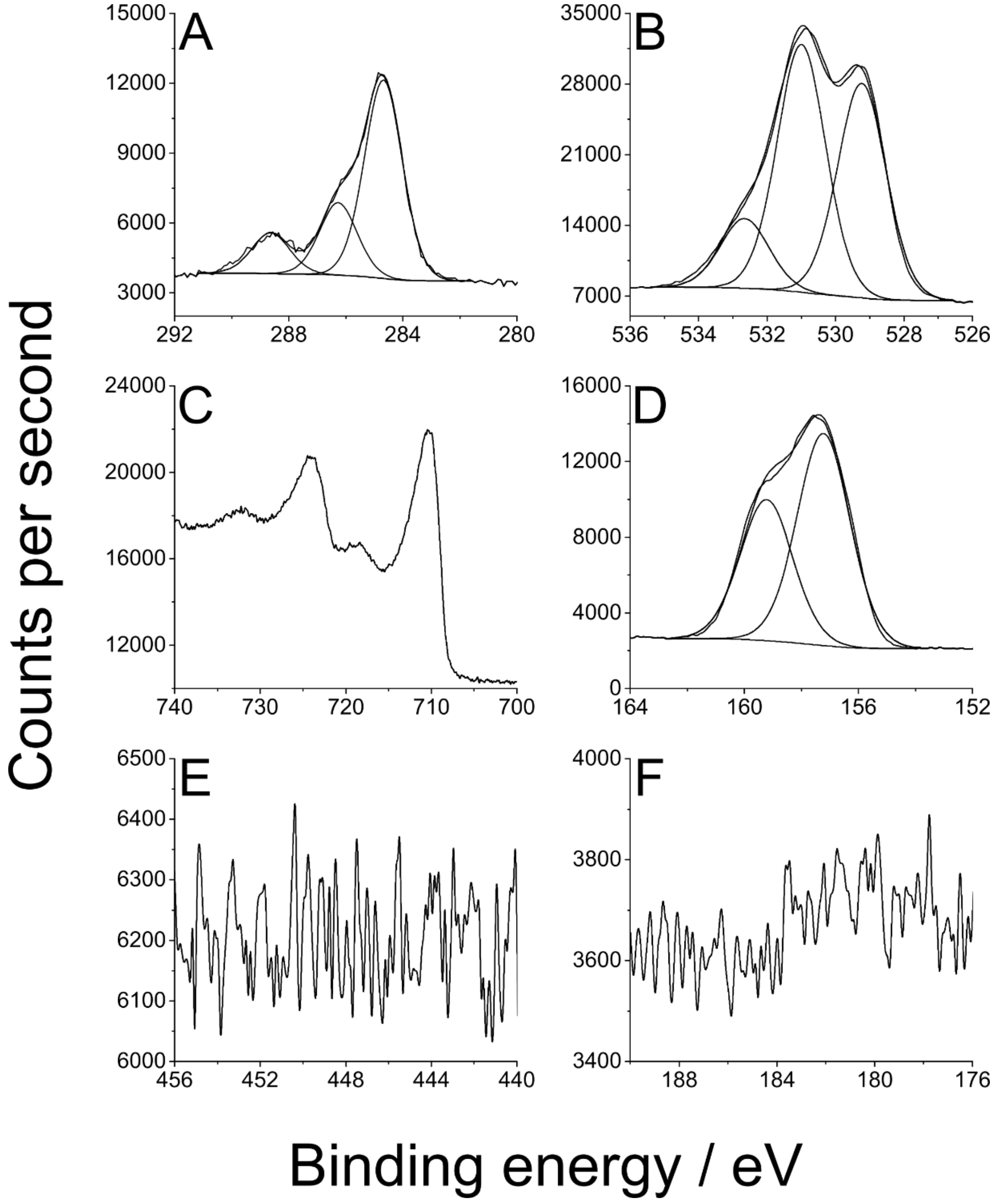

Figure S11: High resolution XPS spectra of the yttrium-iron oxide control sample, showing: A: C1s (3 environments at 284.7 eV, 286.3 eV and 288.6 eV), B, O1s (3 environments at: 529.2 eV, 531.0 eV and 532.7 eV), C: Fe2p (peaks at 710.3 eV ($Fe2p_{3/2}$) and 724.1 eV($Fe2p_{1/2}$)), D: Y3d ((1 environment at 157.2 eV ($Y3d_{5/2}$) and 159.2 eV($Y3d_{3/2}$)) E: In3d and F: Zr3d. Note the complete absence of In and Zr with no signal seen in spectra E and F.

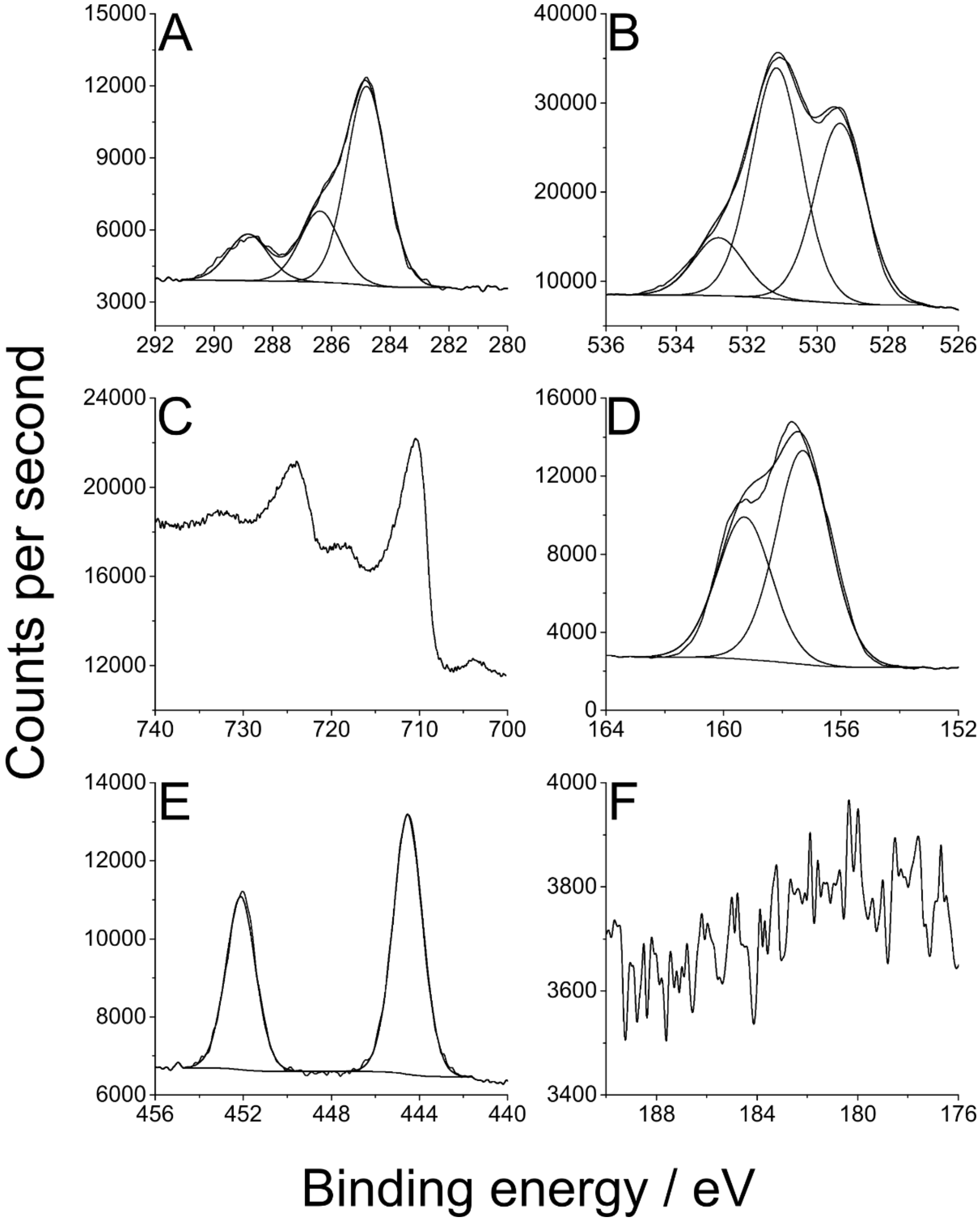

Figure S12: High resolution XPS spectra of the yttrium-iron oxide-In sample, showing: A: C1s (3 environments at 284.8 eV, 286.4 eV and 288.8 eV), B, O1s (3 environments at: 529.4 eV, 531.2 eV and 532.8 eV), C: Fe2p (peaks at 710.4 eV ($Fe2p_{3/2}$) and 724.2 eV($Fe2p_{1/2}$)), D: Y3d (1 environment at 157.3 eV ($Y3d_{5/2}$) and 159.3 eV($Y3d_{3/2}$)), E: In3d (1 environment at 444.5 eV ($In3d_{5/2}$) and 452.1 eV($In3d_{3/2}$)) and F: Zr3d. Note the complete absence of Zr with no signal seen in spectrum F.

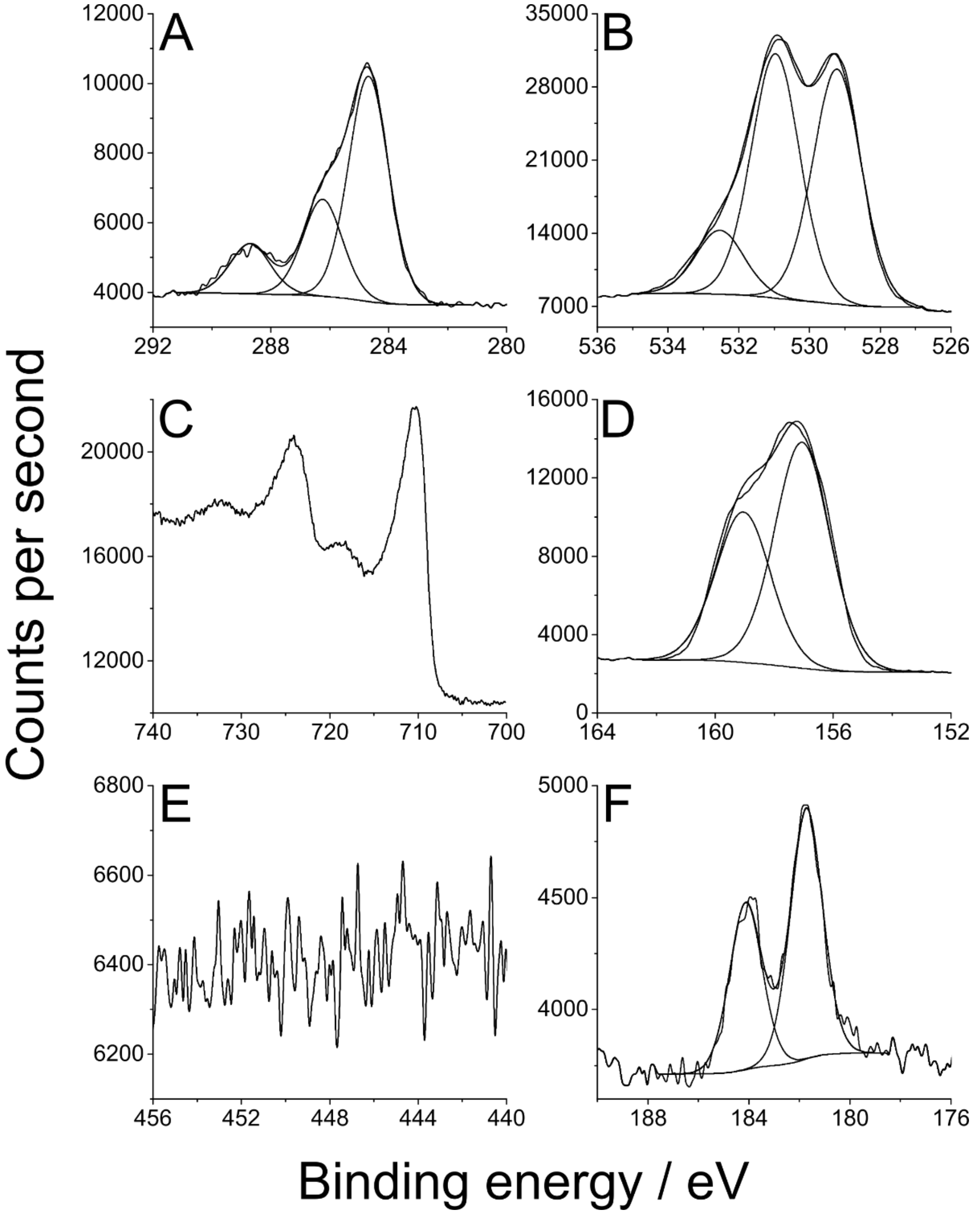

Figure S13: High resolution XPS spectra of the yttrium-iron oxide-Zr sample, showing: A: C1s (3 environments at 284.7 eV, 286.2 eV and 288.7 eV), B, O1s (3 environments at: 529.2 eV, 531.0 eV and 532.5 eV), C: Fe2p (peaks at 710.3 eV ($Fe2p_{3/2}$) and 724.1 eV($Fe2p_{1/2}$)), D: Y3d (1 environment at 157.0 eV ($Y3d_{5/2}$) and 159.0 eV($Y3d_{3/2}$)), E: In3d and F: Zr3d (1 environment at 181.7 eV ($Zr3d_{5/2}$) and 184.1 eV($Zr3d_{3/2}$)).

Additional TEM and EDS data:

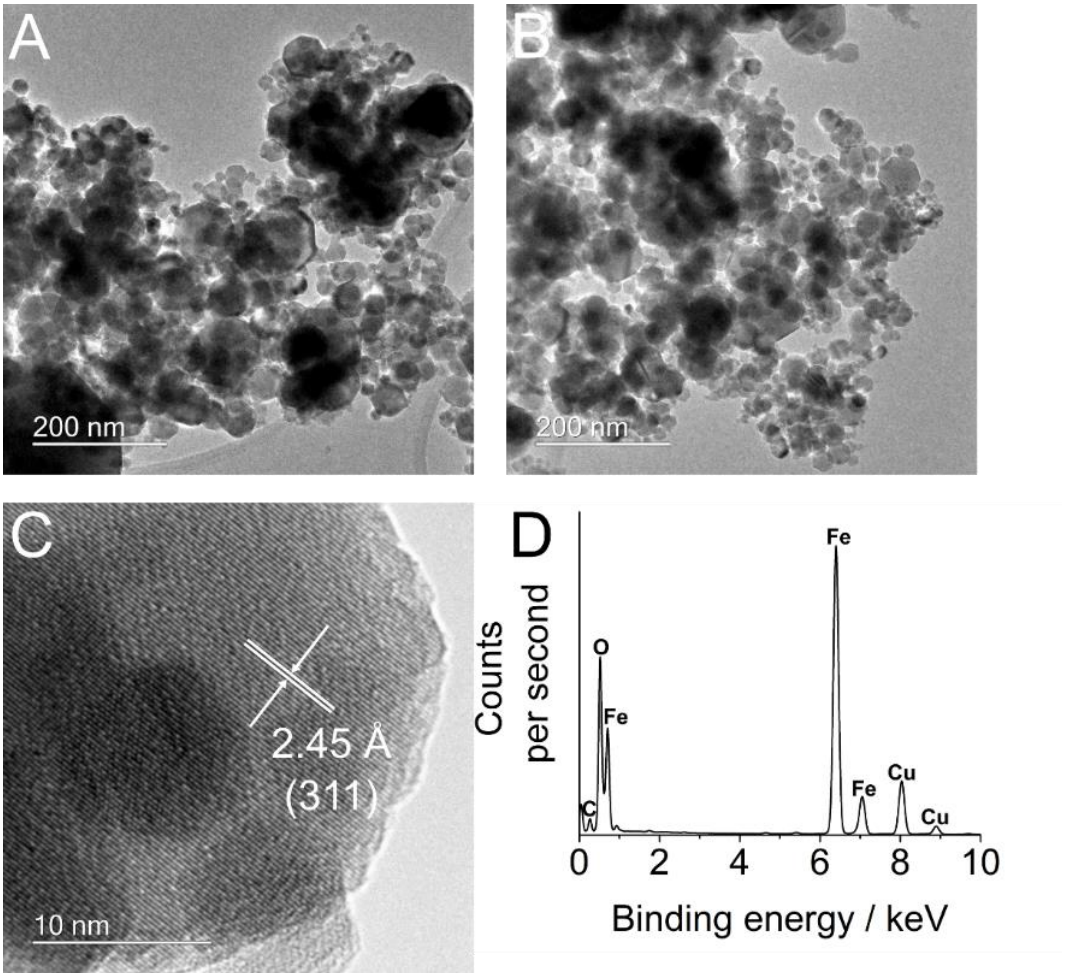

Figure S14: Transmission electron microscope images of the unmodified maghemite (Nanoarc ®) control sample, C shows a high resolution image of an iron oxide particle, assigned as the <311> plane of Fe_2O_3. D is an energy dispersive X-ray spectroscopy (EDS) spectrum of as supplied maghemite (Nanoarc ®) iron oxide nanoparticles. Copper and carbon emanate from the copper TEM grid.

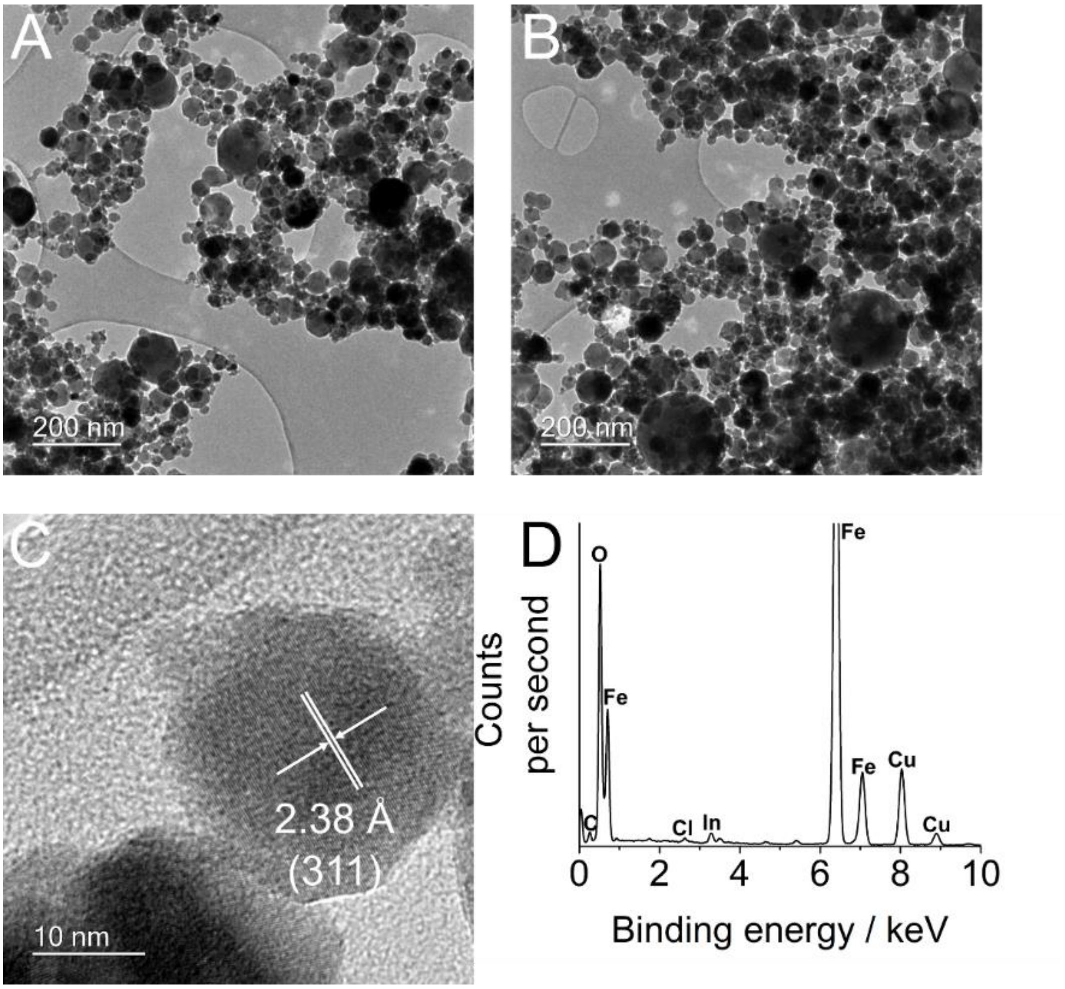

Figure S15: Transmission electron microscope images of the indium-modified maghemite (Nanoarc ®) sample, C shows a high resolution image of an iron oxide particle, assigned as the <311> plane of Fe_2O_3. D is an energy dispersive X-ray spectroscopy (EDS) spectrum of the indium-modified maghemite (Nanoarc ®) iron oxide nanoparticles. Copper and carbon emanate from the copper TEM grid.

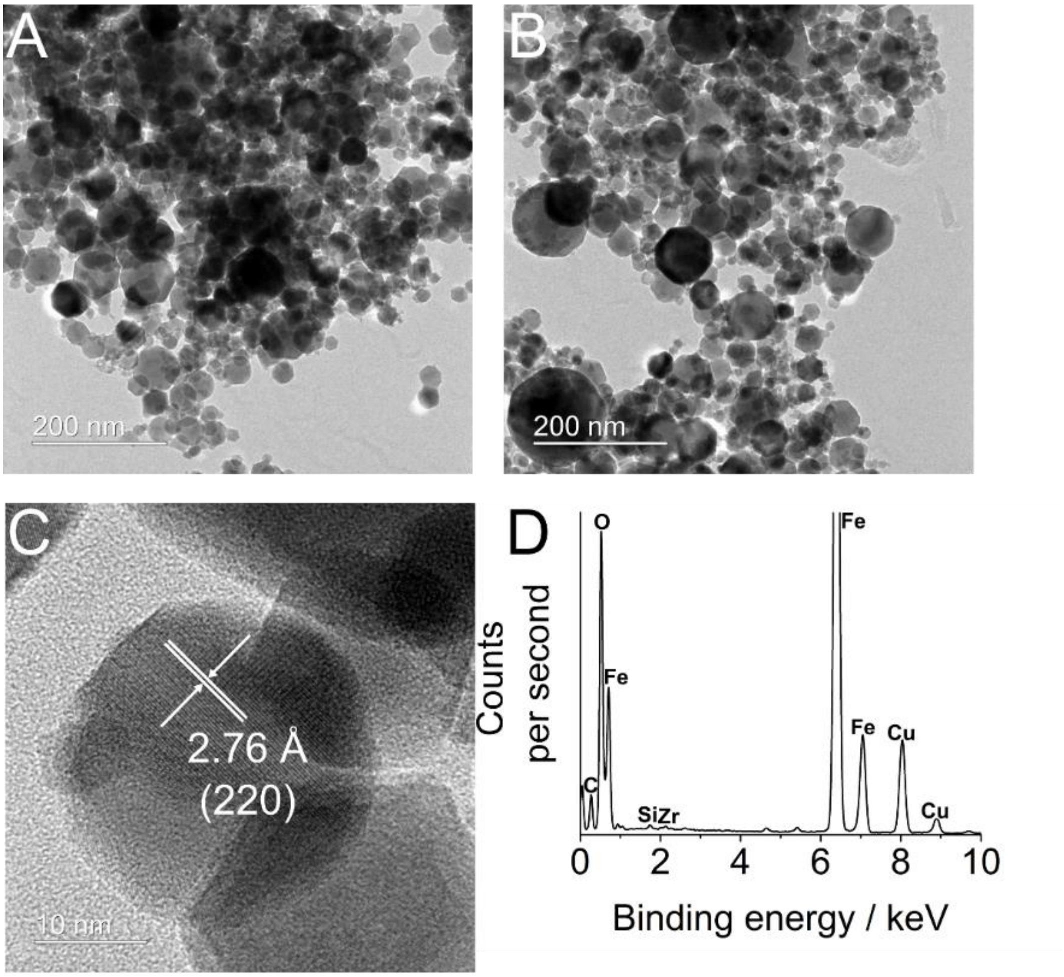

Figure S16: Transmission electron microscope images of the zirconium-modified maghemite (Nanoarc ®) sample, C shows a high resolution image of an iron oxide particle, assigned as the <220> plane of Fe_2O_3. D is an energy dispersive X-ray spectroscopy (EDS) spectrum of the zirconium-modified maghemite (Nanoarc ®) iron oxide nanoparticles. It is noteworthy that the small amount of zirconium present was commensurate with the weak signal observed in XPS (figure S10). Copper and carbon emanate from the copper TEM grid.

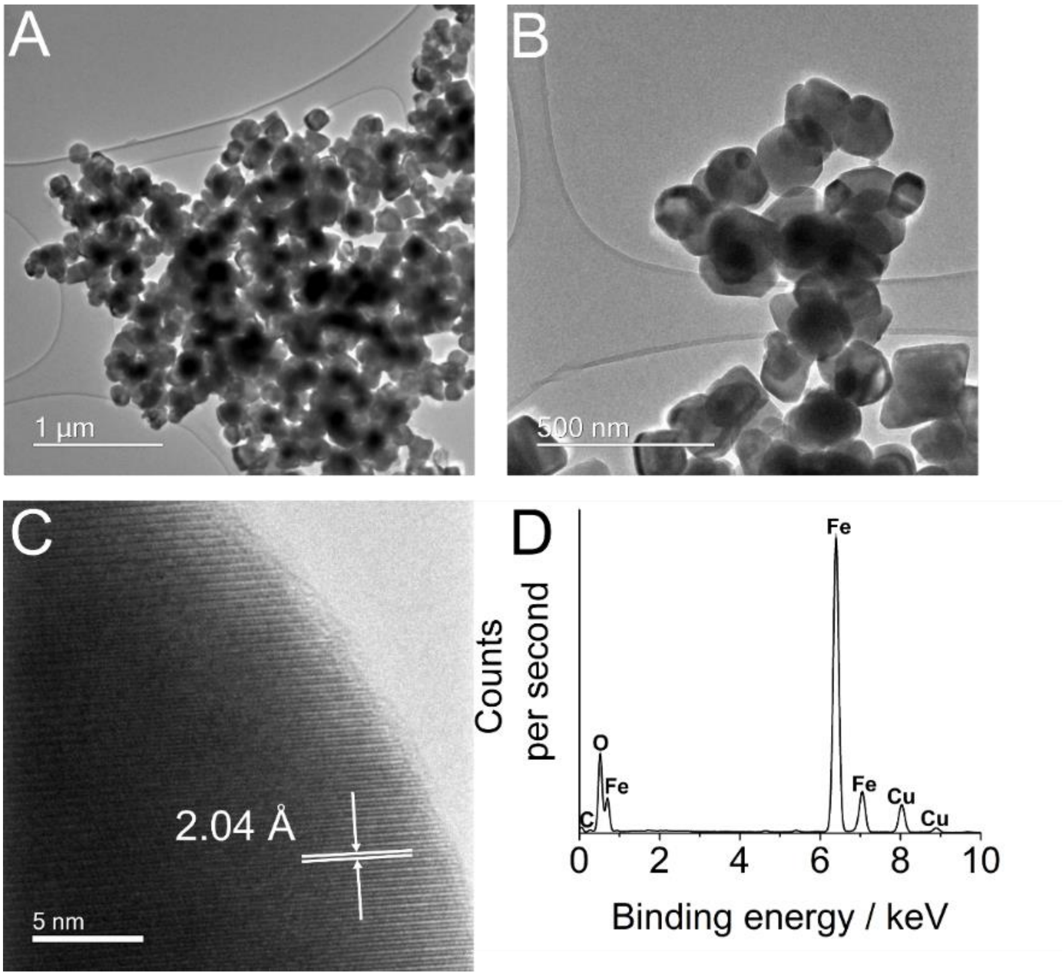

Figure S17: Transmission electron microscope images of the unmodified magnetite control sample, C shows a high resolution image of an iron oxide particle, showing a d spacing of 2.04Å. D is an energy dispersive X-ray spectroscopy (EDS) spectrum of unmodified magnetite iron oxide nanoparticles. Copper and carbon emanate from the copper TEM grid.

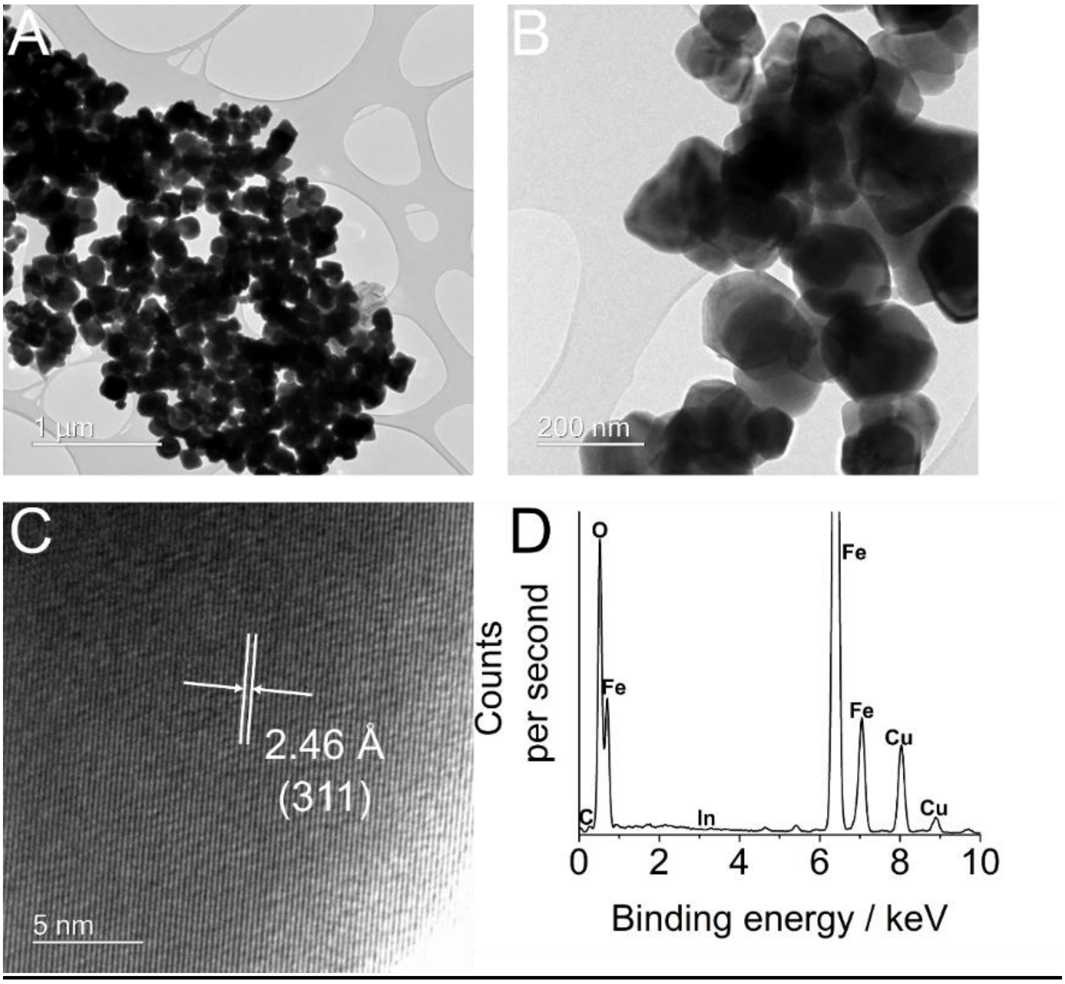

Figure S18: Transmission electron microscope images of the indium-modified magnetite sample, C shows a high resolution image of an iron oxide particle, assigned as the <311> plane of iron oxide. D is an energy dispersive X-ray spectroscopy (EDS) spectrum of the indium-modified magnetite iron oxide nanoparticles. Copper and carbon emanate from the copper TEM grid. Indium was not plentiful here, but presence was confirmed by XPS (figure S6).

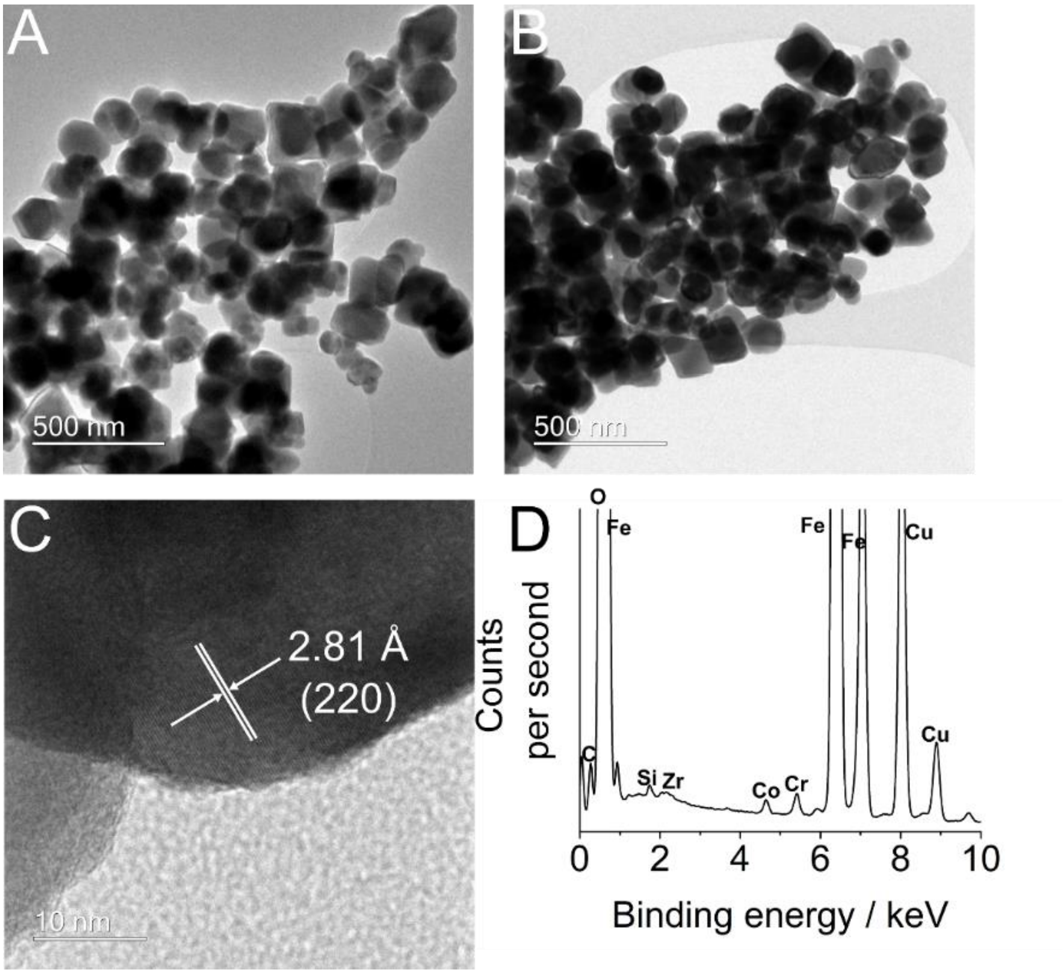

Figure S19: Transmission electron microscope images of the zirconium-modified magnetite sample, C shows a high resolution image of an iron oxide particle, assigned as the <220> plane of iron oxide. D is an Energy dispersive X-ray spectroscopy (EDS) spectrum of the zirconium-modified magnetite iron oxide nanoparticles. Copper and carbon emanate from the copper TEM grid.

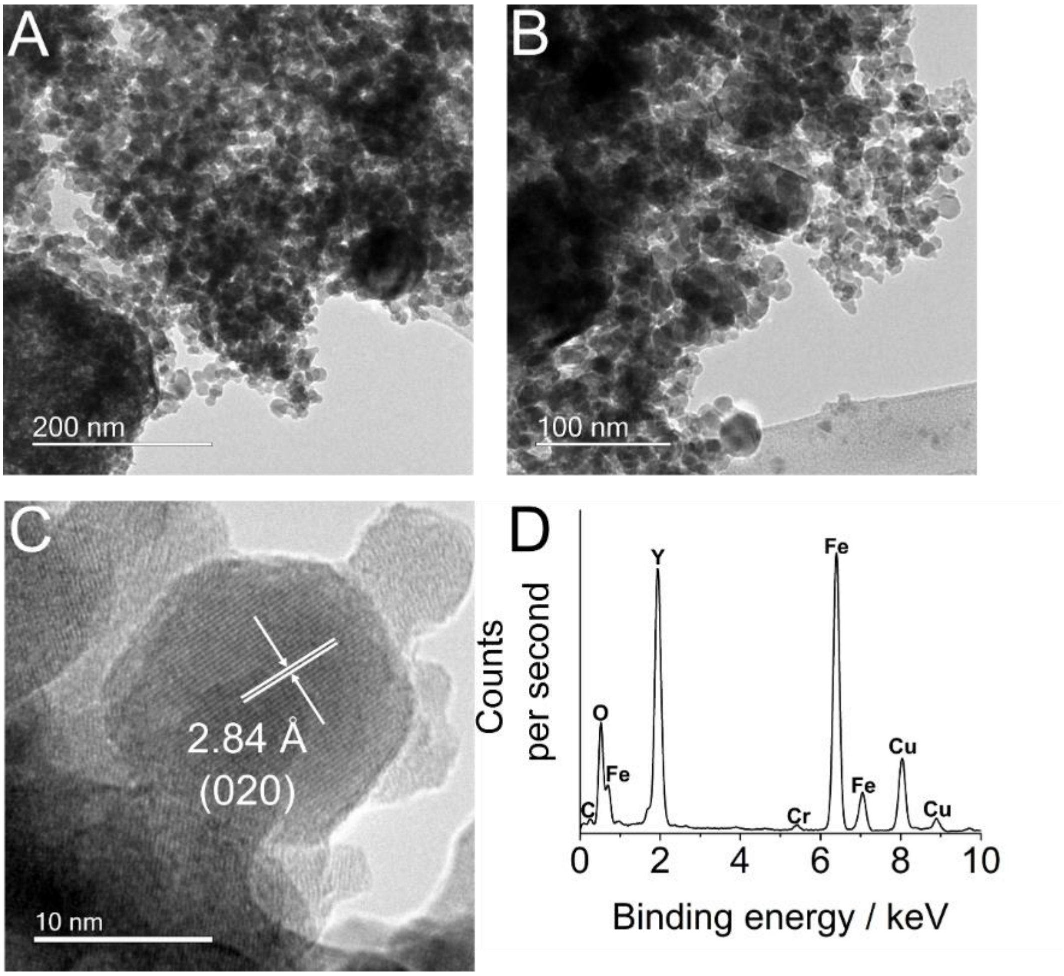

Figure S20: Transmission electron microscope images of the unmodified yttrium-iron oxide ($Y_3Fe_5O_{12}$) control sample, C shows a high resolution image of an $Y_3Fe_5O_{12}$ particle, assigned as the <020> plane of $Y_3Fe_5O_{12}$. D is an energy dispersive X-ray spectroscopy (EDS) spectrum of unmodified $Y_3Fe_5O_{12}$ nanoparticles. Copper and carbon emanate from the copper TEM grid and chromium from the steel TEM goniometer.

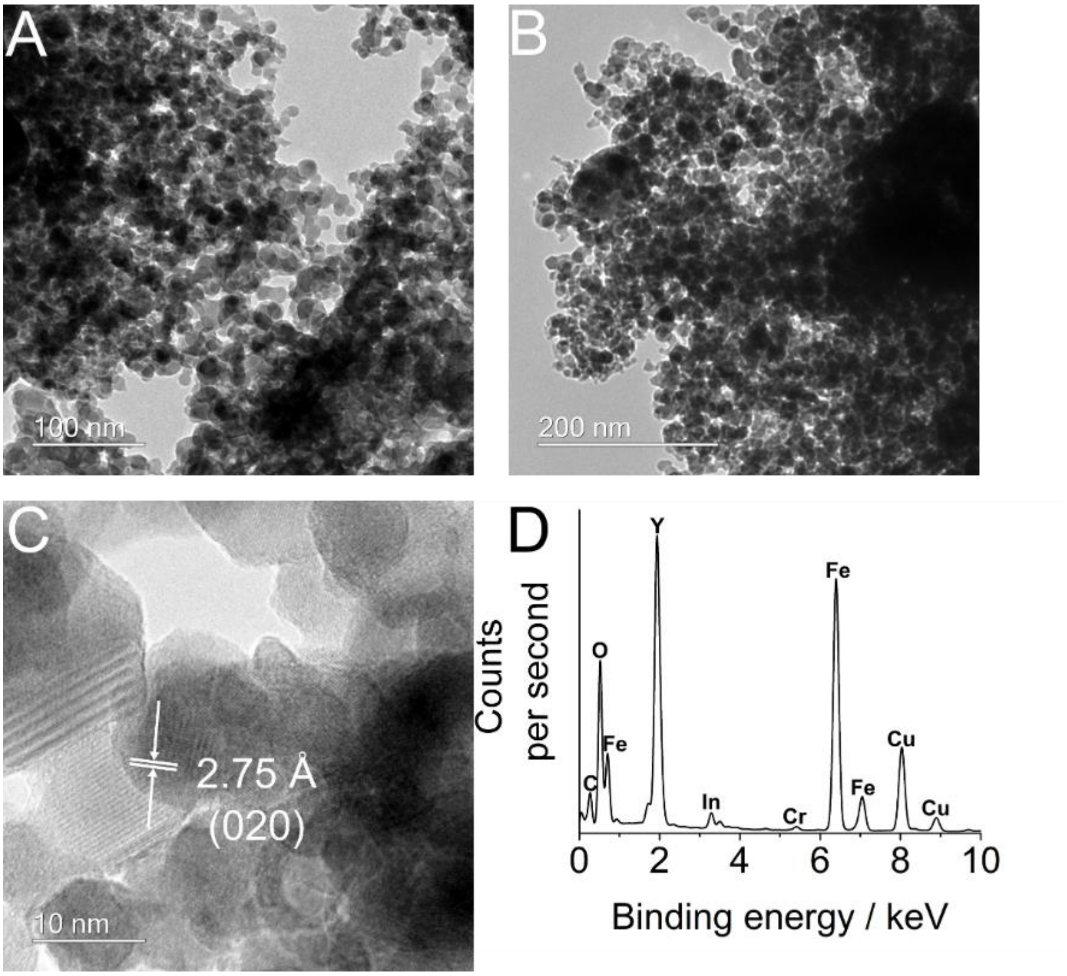

Figure S21: Transmission electron microscope images of the indium modified yttrium-iron oxide ($Y_3Fe_5O_{12}$) sample, C shows a high resolution image of an $Y_3Fe_5O_{12}$ particle, assigned as the <020> plane of $Y_3Fe_5O_{12}$. D is an energy dispersive X-ray spectroscopy (EDS) spectrum of indium modified $Y_3Fe_5O_{12}$ nanoparticles. Copper and carbon emanate from the copper TEM grid and chromium from the steel TEM goniometer.

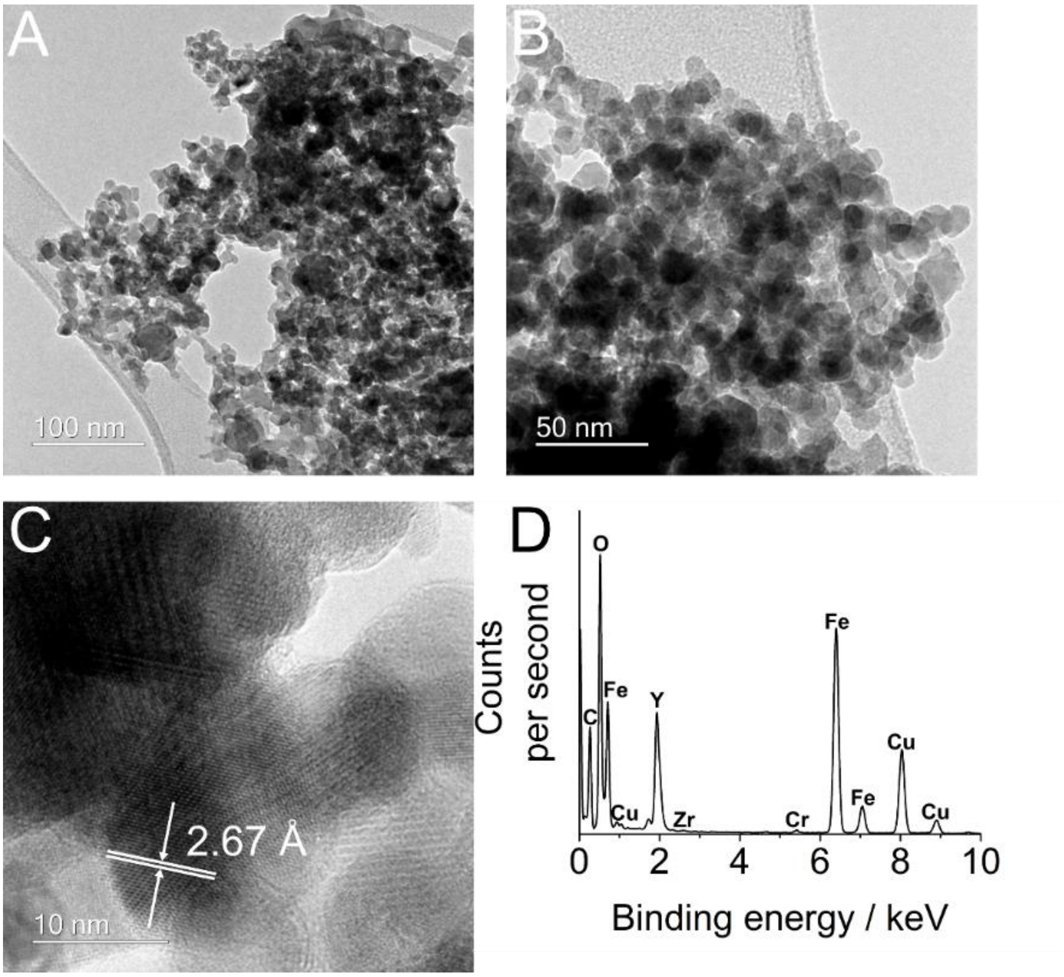

Figure S22: Transmission electron microscope images of the zirconium modified yttrium-iron oxide ($Y_3Fe_5O_{12}$) sample, C shows a high resolution image of an $Y_3Fe_5O_{12}$ particle, showing a d spacing of 2.67Å. D is an energy dispersive X-ray spectroscopy (EDS) spectrum of zirconium modified $Y_3Fe_5O_{12}$ nanoparticles. Copper and carbon emanate from the copper TEM grid and chromium from the steel TEM goniometer.

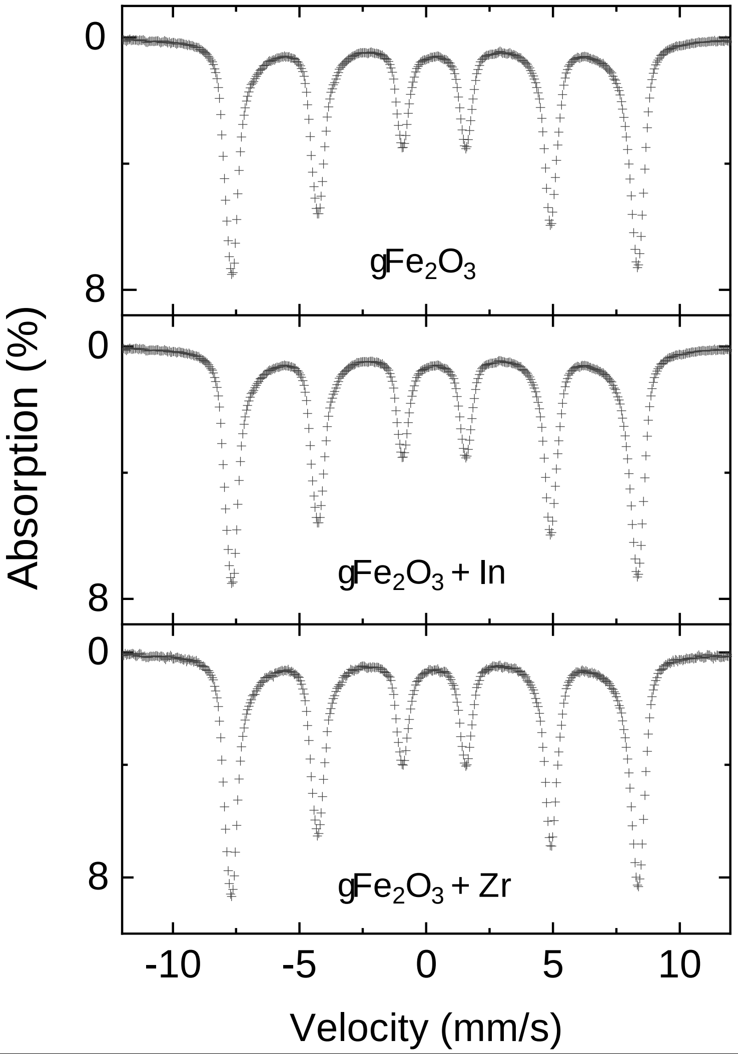

0
8
gFe2O3
0
8
gFe2O3 + In
0
8
gFe2O3 + Zr
Absorption (%)
-10
-5
0
5
10
Velocity (mm/s)

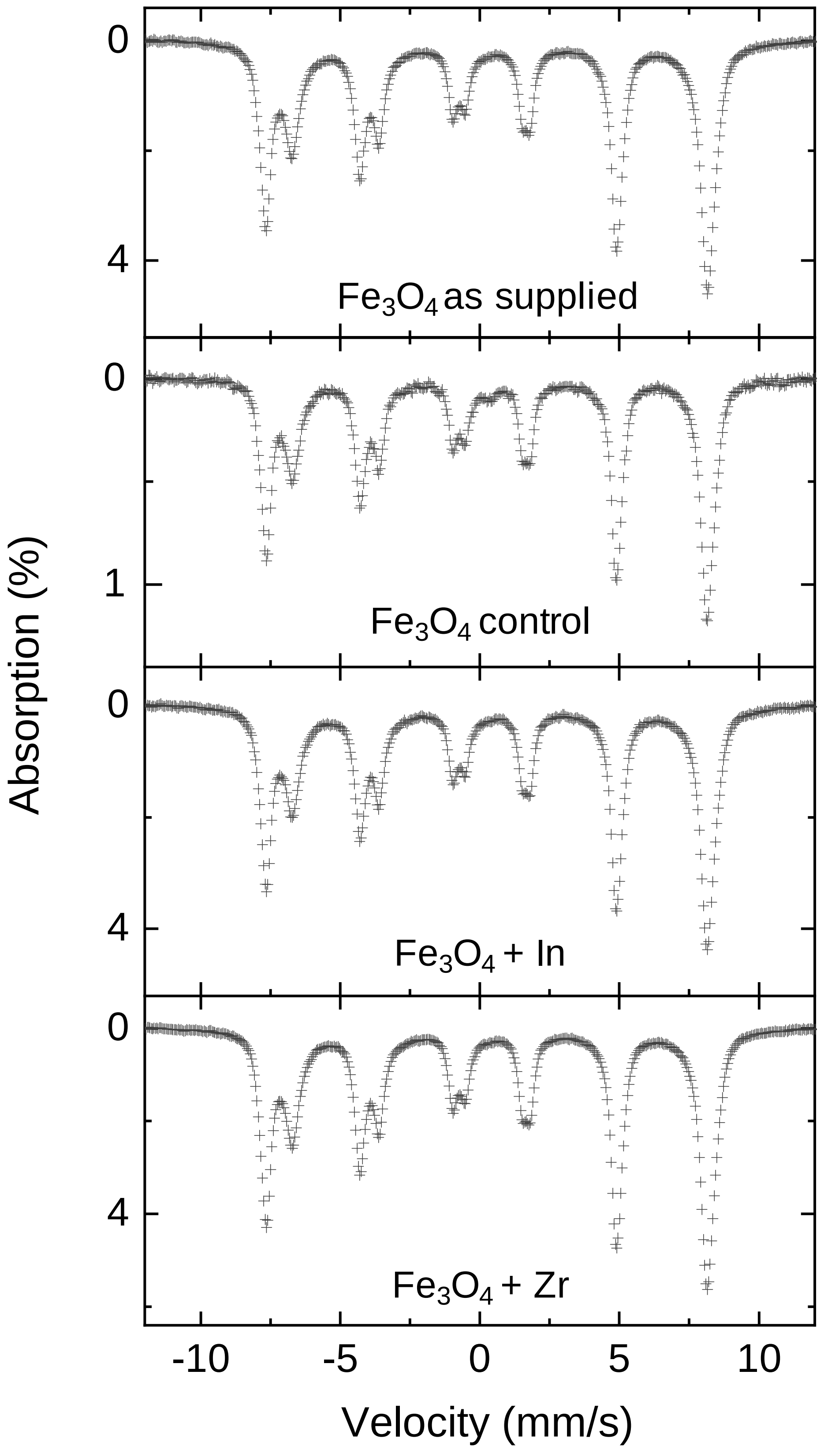

Figure S23. Mössbauer Spectra acquired for Alfa Aesar maghemite (Fe_2O_3) particles, and Sigma Aldrich magnetite/maghemite (Fe_3O_4 / Fe_2O_3) particles before and after heat induced labelling with non-radioactive In and Zr additives. No change in the proportion of Fe atoms in a maghemite and magnetite environment was seen following the labelling reaction. For the magnetite/maghemite particles, the proportion of the particle containing magnetite decreased following heating in the absence of Zr or In additives (from 0.64 to 0.59), however when heated with these the amount remained constant (0.68 to 0.63). It is interesting to note, however, that for the Sigma Aldrich

sample treated in the absence of the metal shows a small feature in the centre of the spectrum (indicated by the arrow) and which is not present in either the untreated sample or those treated with In and Zr. Furthermore, the *wt*.% magnetite content decreases to ≈ 59%, indicating that in the absence of the radio-metal, the treatment process is oxidative, with the metal additive offering some protection against this. From this, we hypothesise that the treatment results in the formation of a very thin surface layer on the surface of the FeO_x nanoparticles.

SQUID magnetometry:

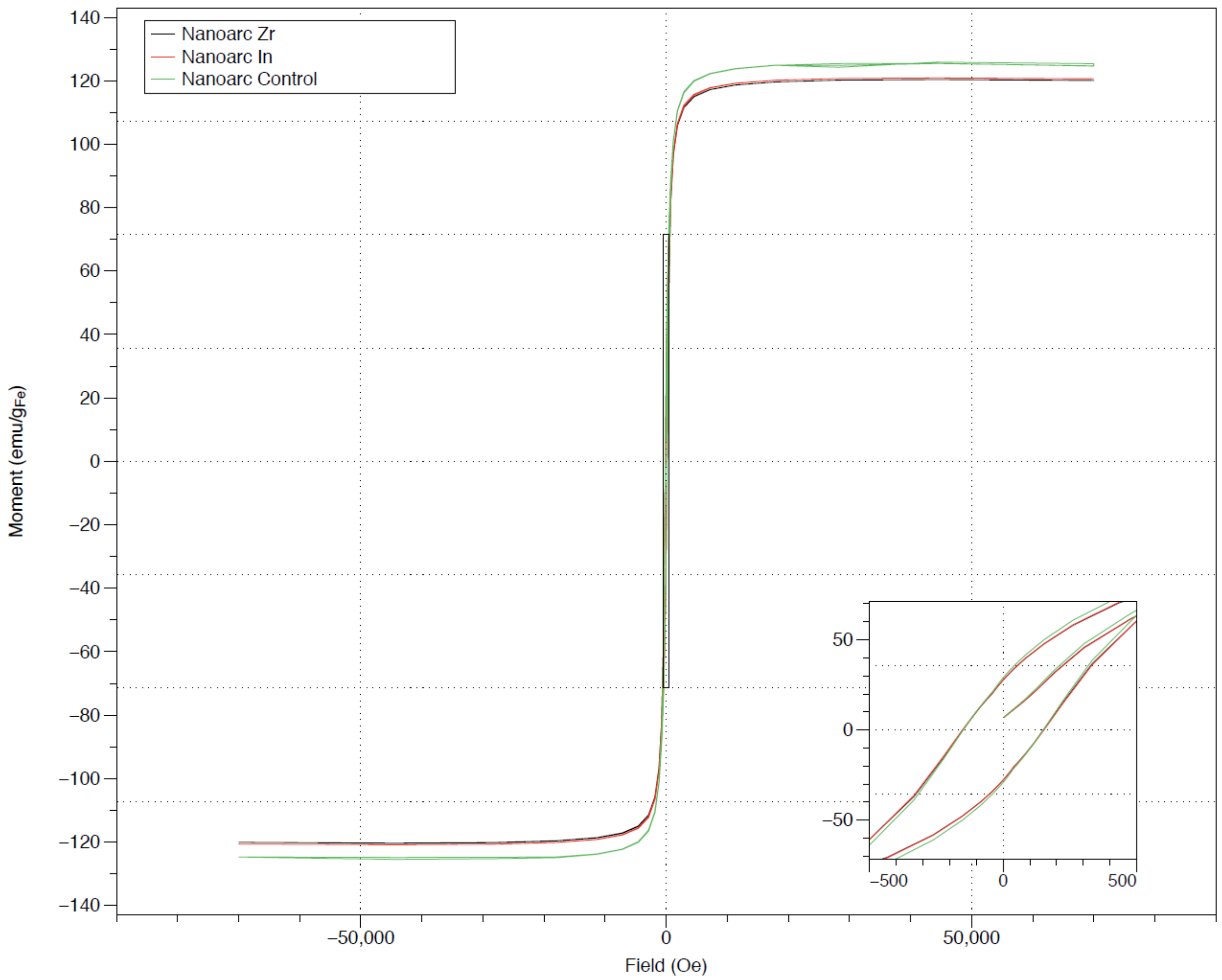

Figure S24. SQUID measurement for maghemite nanoparticles (Nanoarc, Alfa Aesar), modified with $ZrCl_4$, $InCl_3$, or unreacted stock particles (control). Heat induced radiolabelling with In and Zr does not affect magnetic hysteresis properties of the particles including their saturation magnetisation, coercivity, or remanence.

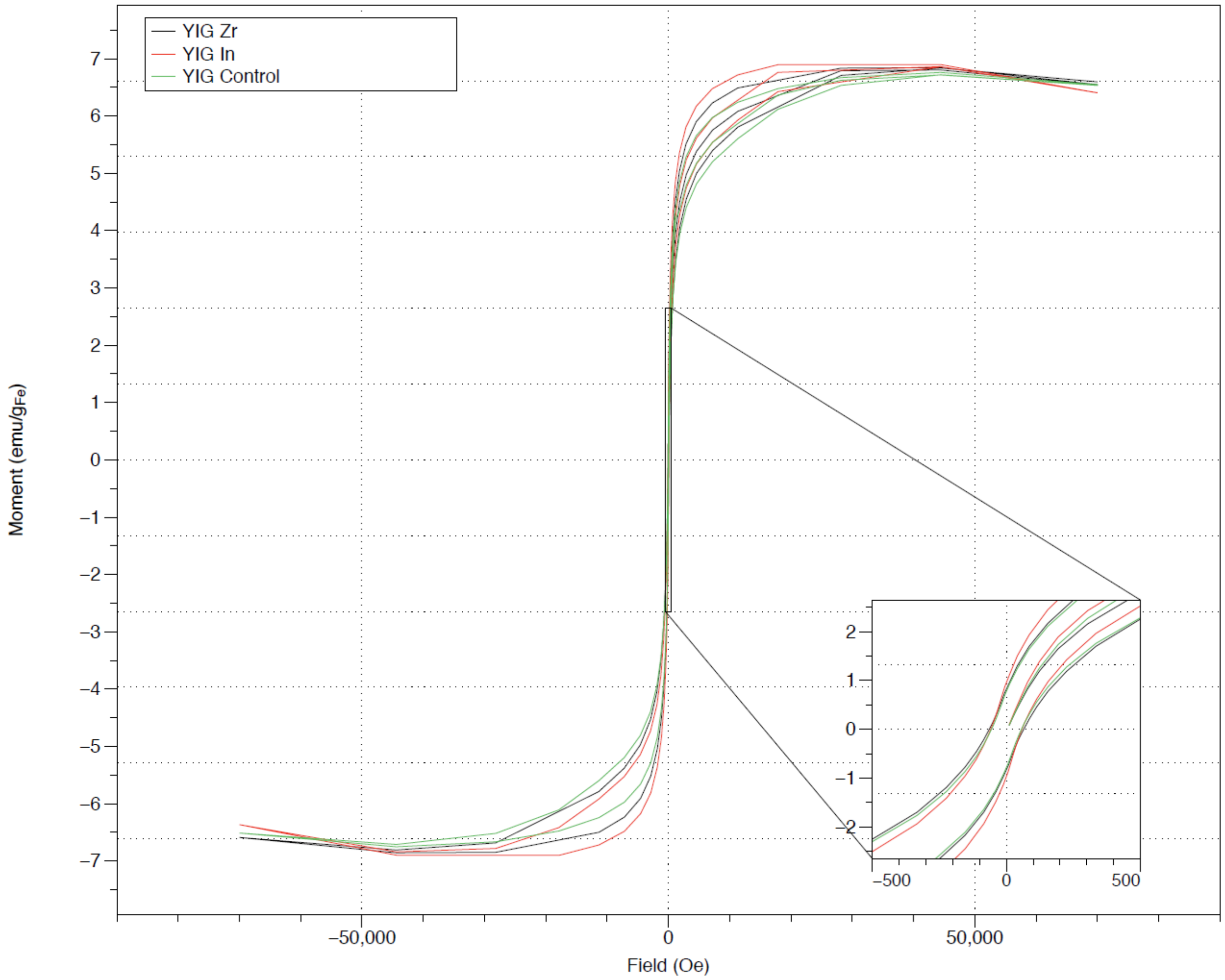

Figure S25. SQUID measurement for yttrium-iron oxide nanoparticles ($Y_3Fe_5O_{12}$), modified with $ZrCl_4$, $InCl_3$, or unreacted stock particles (control). Heat induced radiolabelling with In and Zr slightly affects the coercivity of the particles but not their saturation magnetisation or remanence.

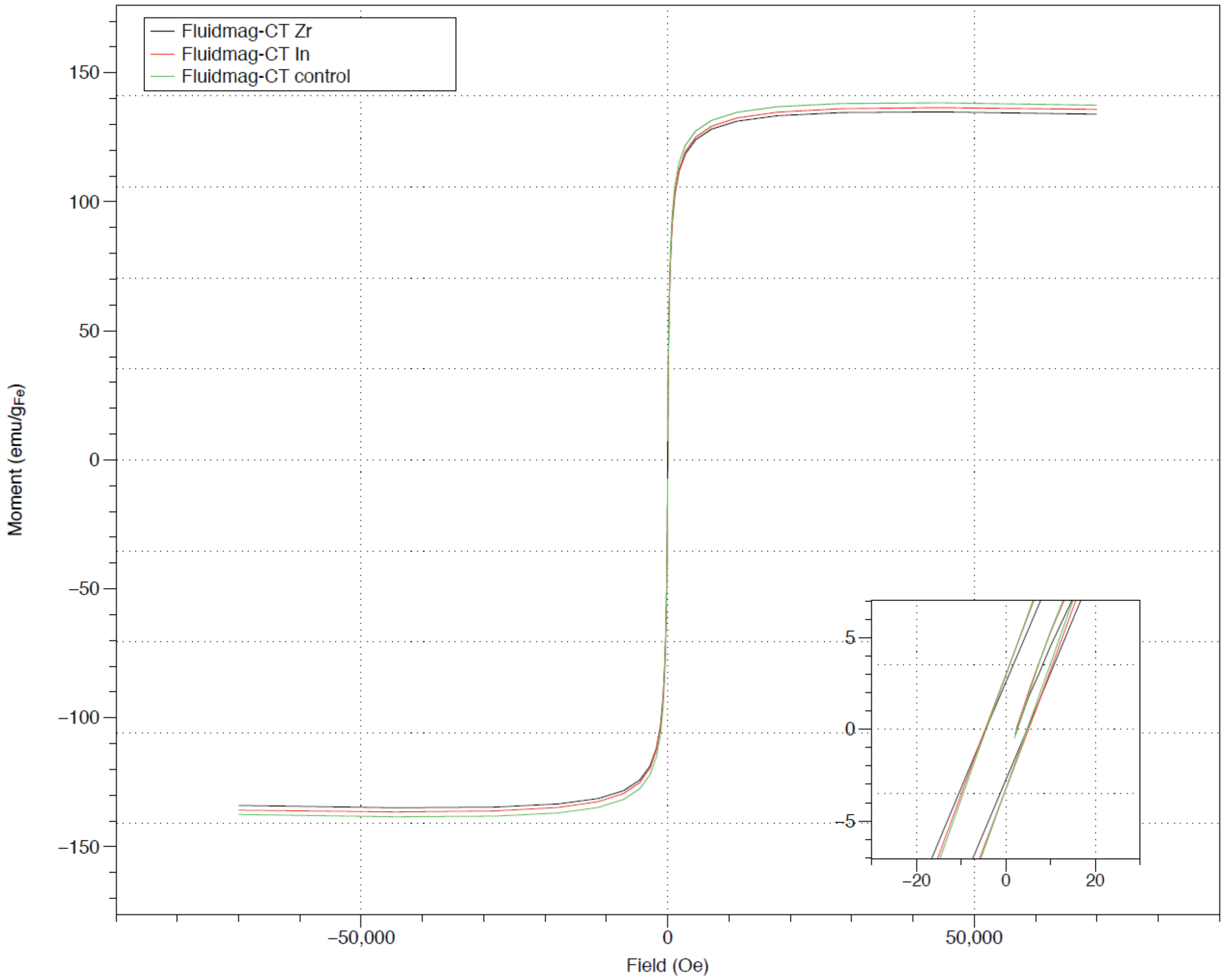

Figure S26. SQUID measurement for FluidMag CT nanoparticles (200 nm, Chemicell), modified with $ZrCl_4$, $InCl_3$, or unreacted stock particles (control). Heat induced radiolabelling with In and Zr does not affect magnetic hysteresis properties of the particles including their saturation magnetisation, coercivity, or remanence.

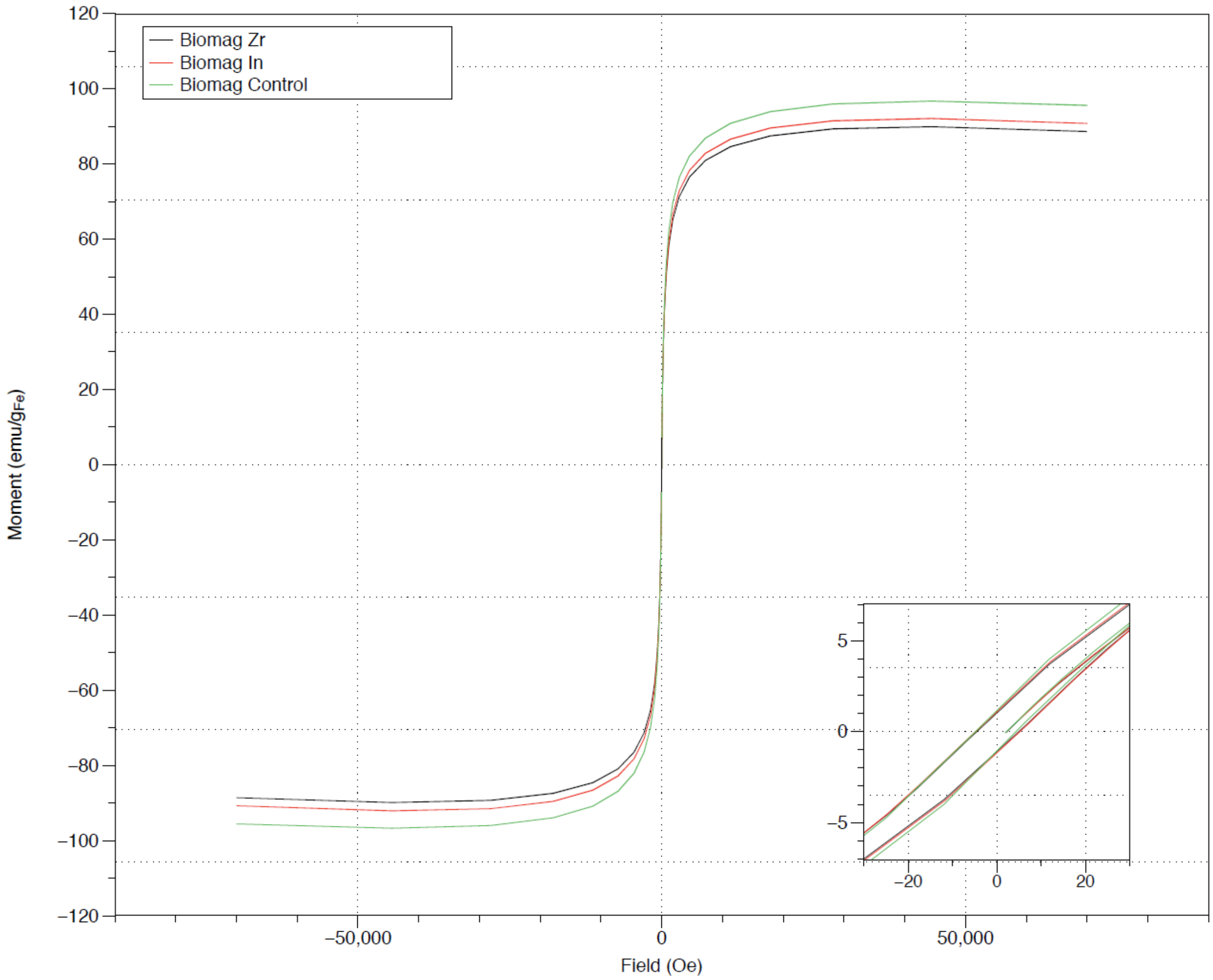

Figure S27. SQUID measurement for Biomag Maxi Carboxyl particles (3 to 12 µm, Bangs Laboratories), modified with $ZrCl_4$, $InCl_3$, or unreacted stock particles (control). Heat induced radiolabelling with In and Zr does not affect magnetic hysteresis properties of the particles including their saturation magnetisation, coercivity, or remanence.

ToF-SIMS:

The Surface location of additives was investigated using time of flight-secondary ion mass spectroscopy (ToF-SIMS), where depth profile measurements were able to confirm surface bound dopants.

Figure S28 shows the indium doped $Y_3Fe_5O_{12}$ nanoparticles. ToF-SIMS analysis shows that the indium is more abundant at the surface and tappers off as etching increases, which is in line with our expectations. In contrast, the Fe stays relatively consistent with a slight increase with sputter time, indicating its dominance in the core of the nanoparticles (as per the AA maghemite Fe_3O_4 sample).

Figure S29 shows the control $Y_3Fe_5O_{12}$ nanoparticle samples. This showed a consistent Fe and Y profile without the presence of any dopants. The AA maghemite control sample showed a consistent Fe profile without any trace of indium.

The analysis of the $Y_3Fe_5O_{12}$ nanoparticles doped with zirconium (Zr) wasn't quite as clear. Firstly, the signal measured for Zr even at the surface showed quite a low response. This may be attributed to the low ionisation efficiency of Zr for this species in this particular matrix. The measurement was further complicated by the fact that Zr^+ has an overlapping mass with the protonated Y fragment (YH^+), where both m/z ~ 89.90. In order to try overcome this overlapping mass limitation, some of the isotopes of Zr were also selected (^{91}Zr, ^{92}Zr and ^{94}Zr) to be monitored to hopefully give a better indication of Zr in the depth profiles (Figure S30). The peak at m/z = 89.90 was confirmed to have a component arising from YH^+ as the previous indium doped yttrium sample also had this peak, but unfortunately no Zr isotopes were present in the spectra. The analysis of the maghemite (Alfa Aesar Nanoarc ®) Fe_3O_4 doped with zirconium was also problematic, potentially again due to the low ionisation efficiency of the Zr. There are many changes occurring on the surface in this transient stage due to the bombardment of the surface in the DC sputter phase.

Despite the difficulty in Zr determination using ToF-SIMS, it is worth noting the surface sensitivity of this technique is significantly higher than the likes of XPS. Zr was successfully detected using XPS (Figure S5-S13).

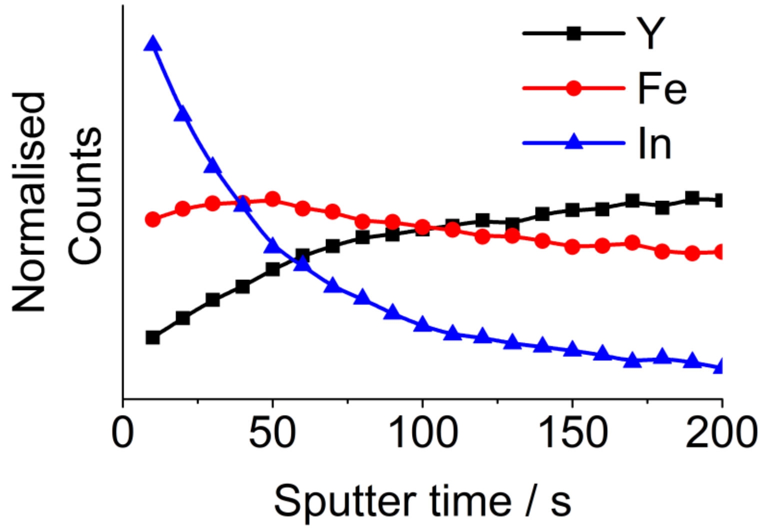

Figure S28. ToF-SIMS analysis demonstrated a sharp decrease in additive concentration with increasing sputter time, indicating its surface bound nature. This trend is observed in both the yttrium-iron oxide ($Y_3Fe_5O_{12}$) nanoparticles and for the indium-doped maghemite (Alfa Aesar Nanoarc) sample.

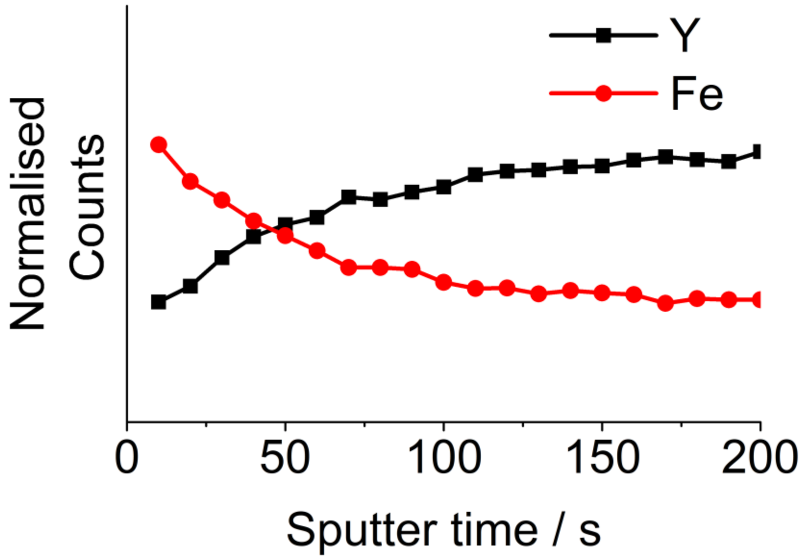

Figure S29: Figure S29 shows the control $Y_3Fe_5O_{12}$ sample. This showed a consistent Fe and Y profile without the presence of any dopants. The AA maghemite control sample showed a consistent Fe profile without any trace of indium.

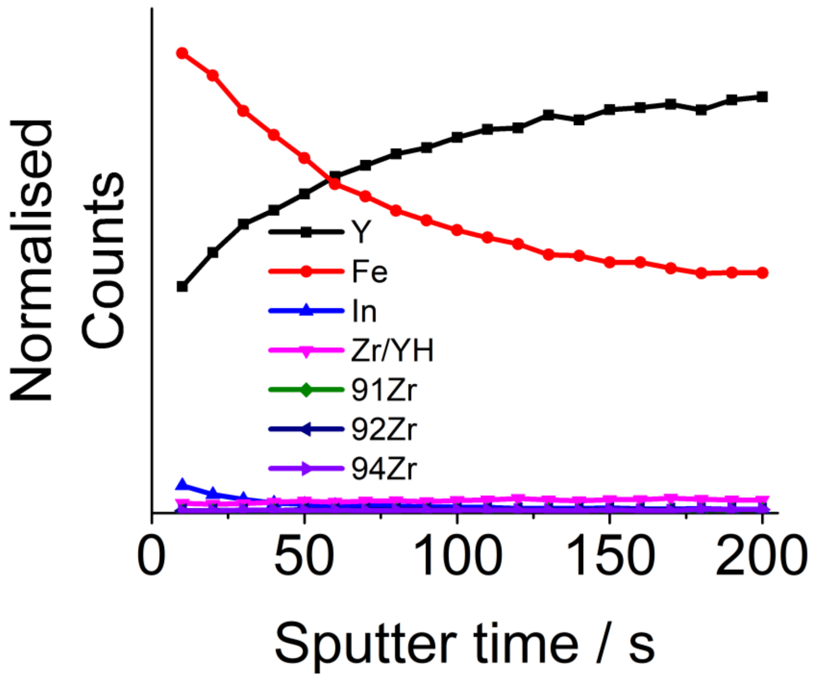

Figure S30: Zirconium-yttrium iron $Y_3Fe_5O_{12}$ particles with added isotopes of Zr (^{91}Zr, ^{92}Zr and ^{94}Zr) showing the minimal overall presence of Zr. Zr was successfully detected using XPS (Figure S5-S13).

[1] J. Fock, L. K. Bogart, D. Gonzalez-Alonso, J. I. Espeso, M. F. Hansen, M. Varon, C. Frandsen, Q. A. Pankhurst, *J. Phys. D: Appl. Phys.* **2017**, *50*, 65005-65005.

[2] K. Lagarec, D. G. Rancourt, *Department of Physics, University of Ottawa, Canada* **1998**.
